# Supplementary material for: A universal all-solid synthesis for high throughput production of halide perovskite
Source: Nat Commun. 2022 Dec 1;13:7399. doi: 10.1038/s41467-022-35122-7 (PMC9715688; doi:10.1038/s41467-022-35122-7)
Supplement: Supplementary file 1 — Supplementary Information [file 41467_2022_35122_MOESM1_ESM.pdf]

Supplementary Information

A universal all-solid synthesis for high throughput production of halide perovskite

Zheng et al.

Supplementary Materials for

A universal all-solid synthesis for high throughput production of halide perovskite

Luyao Zheng, Amin Nozariasbmarz, Yuchen Hou, Jungjin Yoon, Wenjie Li, Yu Zhang, Haodong Wu, Dong Yang, Tao Ye, Mohan Sanghadasa, Ke Wang, Bed Poudel, Shashank Priya, Kai Wang

Corresponding authors. Email: kaiwang@psu.edu (K.W.); sup103@psu.edu (S.P.); bup346@psu.edu (B.P.); aln192@psu.edu (A.N.)

The PDF file includes:

Supplementary Notes 1-4

Supplementary Figures. 1-26

Supplementary Tables 1-5

Supplementary References 1-48

Supplementary Notes

Supplementary Note 1 TRPL average lifetime

We measured the TRPL spectra of thin film, powder, and FAST-$MAPbI_3$ using a 505 nm femtosecond laser as excitation light source and a time-correlated single photon counting detector for signal collection.

Reconvolution

After obtaining the raw data, instrument response function (IRF) spectra was also obtained to correct the spectra of sample of interest. This is because for real lifetime measurements, the IRF, $I(t)$, is not infinitely small. The shape and width of the $I(t)$ are the results from the excitation light pulse (e.g., duration, shape) as well as the response time of the instrument. In typical, the decay of photocarrier can be modeled by exponential model (e.g., a single e.g., $F(t) = Be^{-\frac{t}{\tau}}$, with B being the scalar factor, and τ being the characteristic lifetime, respectively). In certain cases when the characteristic lifetime of the sample falls in the range of IRF, the samples intrinsic attribute can ($F(t)$) deviate from the measured result of ($M(t)$), in a convolutional relationship with IRF involved[1,2]. This relationship can be expressed by the equation of

$$M(t) = \int_0^t I(t') \cdot F(t - t')\, \mathrm{d}t' \quad (1)$$

Hence, through the reconvolution of $M(t)$ by IRF, intrinsic sample performance of $F(t)$ can be accurately quantified.

Fitting Algorithm

For the TRPL fitting process, we utilized the bi-exponential decay function of

$$f(t) = A_1 exp\left(\frac{-t}{\tau_1}\right) + A_2 exp\left(\frac{-t}{\tau_2}\right) + B \quad (2)$$

where τ_1 is the fast decay lifetime component, A_1 is the fast decay amplitude, τ_2 is the slow decay lifetime component, A_2 is the slow decay amplitude, and B is a constant, respectively. Levenberg–Marquardt algorithm (LMA) is used for seeking the best fitting parameters, and evaluated by the parameter of:

$$\chi^2 = \sum_k w_k^2 \frac{[f_k - F_k]^2}{n} \quad (3)$$

Where the w_k is the weighting factor, f_k is the fitting value and F_k is the experimental value, n is the number of free parameters approximately to the number subtracted from the fitted data points by the number of lifetime parameters used in the fitting. χ^2 has a theoretical limit 1.0 for Poissonian distributed and in typical, χ^2 needs to be less than 1.0 to secure a good fitting. Higher order exponential decay can lead to a smaller χ^2, i.e., better fitting. While choosing the decay model also needs to take consideration of the sample condition. In this work, we utilized the bi-exponential decay because it already exhibited a good fit by showing χ^2 of < 0.5. Additionally, for $MAPbI_3$, it has been widely reported both surface trap and bulk defect can lead to relative quick and slow decay component in the TRPL[3]. This well-agreed model is also consistent to the result observed in this work. Hence, we employ the bi-exponential decay function to quantify these underlying attributes of the samples of interest.

Average lifetime

To extract a general photophysical attribute of different samples, we employed the intensity-weighted average lifetime (τ_{ave}) to quantify the results. Since the PL intensity contribution of a component is proportional to the product $A_i\tau_i$, the intensity-weighted average lifetime (τ_{ave}) is defined as[4]

$$\tau_{ave} = \frac{\int_0^\infty t \sum A_i \exp(-t/\tau_i)dt}{\int_0^\infty \sum A_i \exp(-t/\tau_i)dt} = \frac{\sum A_i \tau_i{}^2}{\sum A_i \tau_i} \quad (4)$$

This intensity-weighted average lifetime (τ_{ave}) can also be defined as the average lifetime of a collection of different excited-state populations. The lifetime of each population is weighted by the relative contribution of that population to the total PL. For halide perovskites, both surface trap (fast component) and bulk defect (slow component) could contribute different population to the overall fluorescence. To include both contributions, we use this intensity-weighted average lifetime (τ_{ave}) to quantify the sample quality. The detailed data are shown in **Supplementary Table 1**.

Briefly, for halide perovskites, both surface trap (faster component) and bulk defect (slower component) could contribute different population to the overall fluorescence. As can be seen in the **Supplementary Table 1**, all the three samples of thin-film, powder, and FAST-$MAPbI_3$ show similar ratio of A1 and A2, which makes it easier to directly compare faster and slower lifetime. Along the surface trap-induced decay, the FAST-samples display the longest lifetime of 72.4 nm compared to thin film (30.2 ns) and powder (6.7 ns). This is consistent to the high trap density nature of the powder and indicates the FAST sample has less surface trap detrimental effect compared to the film sample. Similarly, from the perspective of bulk trap induced decay, the FAST sample also shows longest lifetime of 311 ns, compared to 63 and 77 ns of powder and thin film samples, respectively. This also suggests the high quality of the FAST sample in bulk. While for single crystal sample, as the ratio of A1 and A2 is different from the other three samples, it is hard to directly compare the τ_1 and τ_2. Hence, the τ_{ave} can be a more proper figure-of-merit to evaluate the lifetime. Apparently, single crystal sample shows a smallest value of 20.7 ns. This is more likely due to the presence of hypothetical solvent impurity, surface microstructure and point defect as discussed in previous text in this response file.

Supplementary Note 2 Trap density and hole mobility

To investigate the trap density and charge carrier mobility of the samples, we utilized the space-charge-limited current (SCLC) method by measuring the dark I-V curve on the hole-only device of Au/FAST- $MAPbI_3$/Au. The hole-only device was fabricated by sequential deposition of 100 nm thick Au layers on the both sides of FAST-$MAPbI_3$ sample. The active area (S) is defined to be 0.56 cm^2 and the thickness (L) of FAST-$MAPbI_3$ disk is 1.33 mm. The I-V curve is listed as **Fig. 2D** in the main text, where there are three regions: in the low-bias region, a linear relationship between current density and voltage is observed which is corresponding to an ohmic response; as the voltage develops the current density shows a nonlinear increase which is corresponding to the trap-filling due to the injected charge carriers; at higher voltage region, the current increases quadratically along with the voltage, which is usually termed by "Child regime" that is typically used to describe the space-charge-limited current (SCLC) in a plane-parallel *vacuum* diode with the dependence of voltage (three-halves power)[5].

In the trap filling region (or trap-filled limit (TFL) regime), the traps in the material have all been filled prior to the application of voltage. There is a voltage threshold of V_{TFL} (noted in **Fig. 2D**) for current flow, which is because that before applying the voltage there already exist un-neutralized charges at the traps which will further prevent the electron injection at the electrode. It is thus necessary for the charges to overcome this repulsion by a voltage of V_{TFL}. The mathematical treatment of the TFL law can be referred to a prior paper[6]. In consequence, V_{TFL} can be expressed by the equation of:

$$V_{FTL} = \frac{e n_{trap} L^2}{2\varepsilon_0 \varepsilon} \tag{5}$$

Where the e is the elementary charge, ε_0 and ε_r are the relative permittivity of free space and perovskite, n_{trap} is the trap density, and L is the sample thickness, respectively. Then the trap density can be obtained from equation. We calculated the result to be 5.4×10^{10} cm^{-3}, for the FAST-$MAPbI_3$ sample, which is comparable to those of single-crystals (**Fig. 2E**) and *ca.* 5-order of magnitude lower than those in solution-processed polycrystals (**Fig. 2E**).

In the Child region, the Child behavior does not generally apply to a semiconductor/insulator in a single-carrier device. And the Mott–Gurney law[7,8] is used to quantify the J-V behavior in such situation. Considering the perovskite disk of thickness of L, the current density voltage relationship can be described by the equation of:

$$J = \frac{9}{8}\varepsilon_0 \varepsilon_r \mu \frac{V^2}{L^3} \tag{6}$$

Where ε_0 and ε_r are the relative permittivity of free space and perovskite, V is the voltage and J is the current density. The charge carrier mobility can be calculated by the equation of:

$$\mu = \frac{8JL^3}{9\varepsilon_0 \varepsilon_r V^2} \tag{7}$$

The hole mobility of the FAST-$MAPbI_3$ disk was calculated to be 1.7 $cm^2V^{-1}s^{-1}$, which is also similar to the values of single-crystal samples (**Fig. 2E**) and shows great consistence to its low trap-density feature.

Supplementary Note 3 Decomposition mechanism of Cs_2SnI_6 and residual CsI in FAST sample.

The Cs_2SnI_6 perovskite is believed to exhibit higher air stability compared to other Sn^{2+}-based Pb-free halide perovskites because of the oxidation state of Sn^{4+} in the lattice. Prior reports demonstrated that the Cs_2SnI_6 is stable in the ambient of low relative humidity (RH) (30–50% RH), exhibiting no obvious decomposition over two months[9]. Nevertheless, the CsI can be present in the thin film sample rapidly due to high RH or aqueous conditions. Prior studies report a threshold RH of 80%[10], above which the Cs_2SnI_6 could decompose into CsI and SnI_4, followed by sequential reaction to form $Sn(OH)_4$ by moisture adsorption. The process can be expressed by:

$$Cs_2SnI_6 \leftrightarrow SnI_4 + CsI \qquad (8)$$

$$SnI_4 + H_2O \leftrightarrow Sn(OH)_4 + HI \qquad (9)$$

High RH will push the proceeding of **Supplementary Equation 9** to right side, this will facilitate the proceeding of **Supplementary Equation 8** to right side. As a result, CsI will be left. Particularly for nanoparticles (or powder sample of Cs_2SnI_6 obtained from BM process), multiple defects can be presented at the particle surface. These dislocation defects can be further developed in to 'etch pits' which accelerate the crystal degradation.

Hence, before consolidation into bulk sample by FAST, the Cs_2SnI_6 powders (nanocrystals) provides many active sites ('etch pits') allowing fast reaction with moisture. This is the reason why even using stoichiometric precursors, the FAST-Cs_2SnI_6 still has the CsI peaks (they were formed before the FAST). Only by adding extra SnI_4 as sacrifice agent, the final sample did not show the CsI peaks. In the meantime, we also track the degradation of the after FAST samples. After FAST, we observed a higher air stability of the consolidated samples (than the powder sample), which is due to that the compact disk sample has less such nanoscopic defects, and consequently lower probability of degradation.

Supplementary Note 4 Lattice Thermal Conductivity (κ_l) Calculation

Lattice thermal conductivity (κ_l) can be calculated via subtracting the electronic contribution (κ_e) from the total thermal conductivity (κ), by the equation of[11]:

$$\kappa_l = \kappa - \kappa_e \qquad (10)$$

And κ_e can be estimated from the Wiedemann-Franz relation[12], through the equation of:

$$\kappa_e = L\sigma T \qquad (11)$$

Where L is the Lorenz factor, σ is the electrical conductivity, and is T is the temperature. Direct measurement of L requires a high mobility and remains difficult thus L is typically estimated either as a constant (i.e., 2.44 × 10^{-8} WΩK^{-2} in case of metal), or via a transport model (e.g., single parabolic band (SPB) model that can be obtained by solving Boltzmann transport equations-to experimental data). Under this condition, both Lorenz factor (L) and Seebeck coefficient (S) are the functions of reduced Ferimi level (or reduced chemical protentional (η_F) and carrier scattering factor (r).

(i) For Seebeck coefficient (S): starting from Fermi-Dirac distribution (considering one-type carrier transport and for a parabolic band with r being a scattering parameter, there is $\tau\,(E) = \tau_0 E^r$, where τ_0 is a constant), one can derive to the following equation set[13]:

$$S = \frac{k_B}{e}\left[\eta_F - \frac{(i+1)F_i(\eta_F)}{i\cdot F_{i-1}(\eta_F)}\right] \qquad (12)$$

Where S is the Seebeck coefficient, k_B is the Boltzmann constant, e is the elementary charge, i is relevant to scattering parameter by $i = r + 3/2$, and η_F and $F_i(\eta_F)$ are the reduced Fermi level and integration respectively and can be determined by:

$$\eta_F = \frac{E_F - E_C}{k_B T} \qquad (13a)$$

$$F_i(\eta_F) = \int_{\mathrm{E}_C=0}^{\infty} \frac{x^i}{1+\exp\,(x-\eta_F)}\,dx \qquad (13b)$$

Where E_F is the Ferimi level, and E_C is a cut-off energy level below which all the electrons are completely blocked from participating in conduction.

(ii) For Lorenz factor (L),

$$L = \left(\frac{k_B}{e}\right)^2 \left[\frac{(i+2)F_{i+1}(\eta_F)}{i\cdot F_{i-1}(\eta_F)} - \left[\frac{(i+1)F_i(\eta_F)}{i\cdot F_{i-1}(\eta_F)}\right]^2\right] \qquad (14)$$

Under condition of acoustic phonon scattering-dominated electrical transport, $r = -1/2$. The L, η_F can be calculated from measured S.

For simplicity, we estimate $\frac{(i+1)F_i(\eta_F)}{i\cdot F_{i-1}(\eta_F)}$ to be -2η_F (detailed calculation can be referred to ref.[13]'s appendix). Furthermore, it should be noted that SPB model with acoustic phonon scattering (SPB-APS) is already an approximation. Accurate integration calculation of $\frac{(i+1)F_i(\eta_F)}{i\cdot F_{i-1}(\eta_F)}$ requires high calculation complexity, and thus we employ the empirical formula from Kim et al.[14] to calculate the L. **Figs. S22A & S22B** shows the plot of temperature dependent reduced Fermi level and Lorenz factor, respectively.

Supplementary Figures

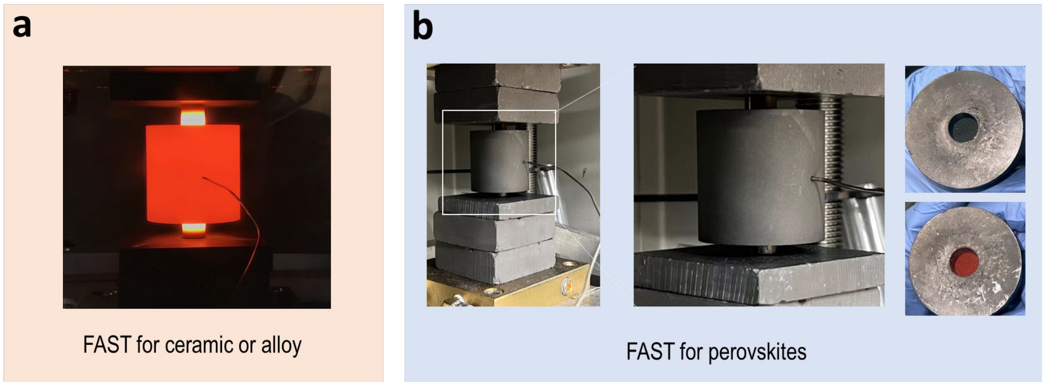

Supplementary Fig. 1 Graphite dies during FAST heating for different materials. a Photograph showing the FAST for ceramics or metallic alloys where the graphite is heated to above 900 °C. **b** In contrast, the synthesis of halide perovskite can use lower temperature FAST (200-500 °C). The photo shows a real-time FAST synthesis of different perovskites and perovskite powder samples ($MAPbI_3$ and $Cs_3Bi_2I_9$) mounted within the die.

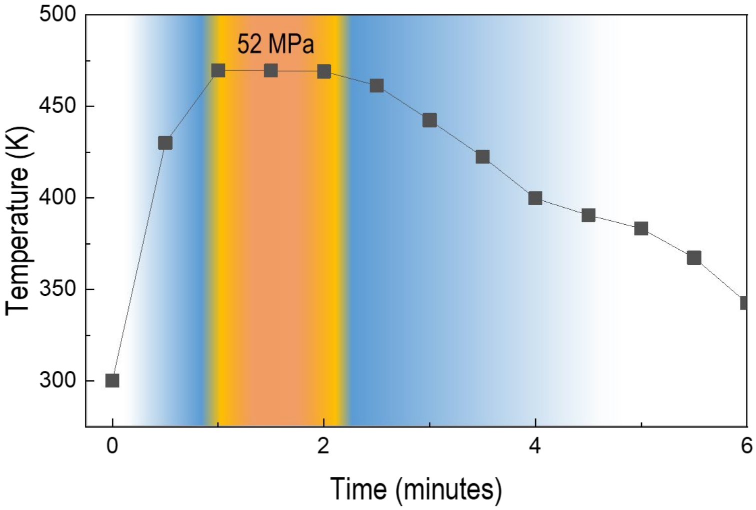

Supplementary Fig. 2 Exemplified temperature - time dependence curve for FAST-$MAPbI_3$. The heating process takes less than 2 min and cooling ramp takes slightly more time (e.g., 3-5 min). Notably, all the parameters can be adjusted as the FAST set up is customized in lab, extra pressure or temperature range, or more precise control can be incorporated as well.

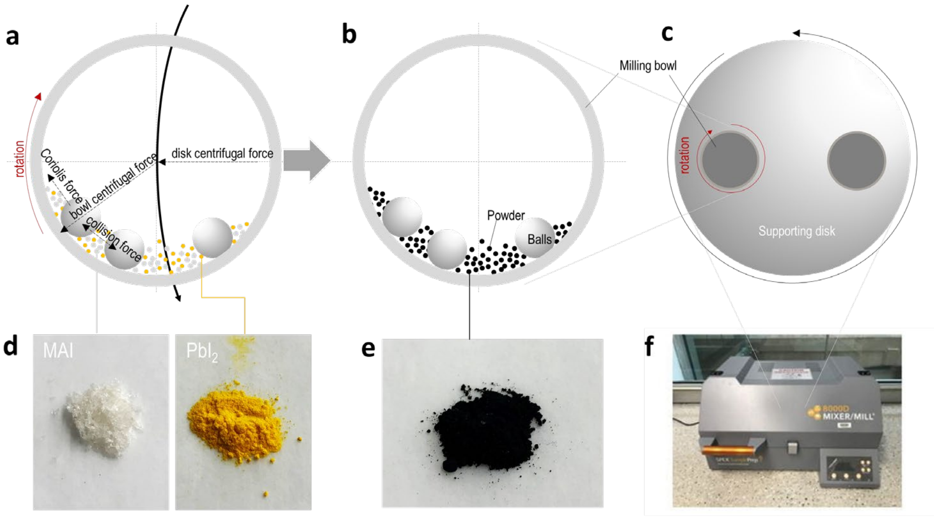

Supplementary Fig. 3 Ball milling (BM) process of $MAPbI_3$. (a-c) Working principles of BM: the bowls and the supporting disk (holder) rotates in opposite directions around their center points. **a** Scheme showing mechanical forces in the bowl. There are multiple types of force executing on the balls including collision force, bowl centrifugal force, Coriolis force, and holder centrifugal force, which can further apply to the precursor powder. **b** Scheme showing the conversion into perovskite powder after BM. **c** The supporting disk on which the milling jars (bowls) are mounted. It goes in opposite rotation direction to the jars due to the different rotational axes of the main disk and the milling jars. This reversed rotation creates a 'D shape' movement of balls inside the jar under the influence of Coriolis and centrifugal forces. In this scenario, kinetic energy within the jars is supposed to increase and lead to high energy ball-to-ball and ball-to-wall impacts, which will effectively grind and blend the perovskite precursors. **d** Pictures showing the precursor powders of MAI and PbI_2 before BM. **e** Picture showing the black $MAPbI_3$ powder after BM. The easy reaction between precursors, plus the high kinetic energy provides the quick conversion from precursors into the black powder. We also carry out the material characterizations on the BM-synthesized powder of $MAPbI_3$, which will be discussed in the next figure. **f** The photo of the BM setup.

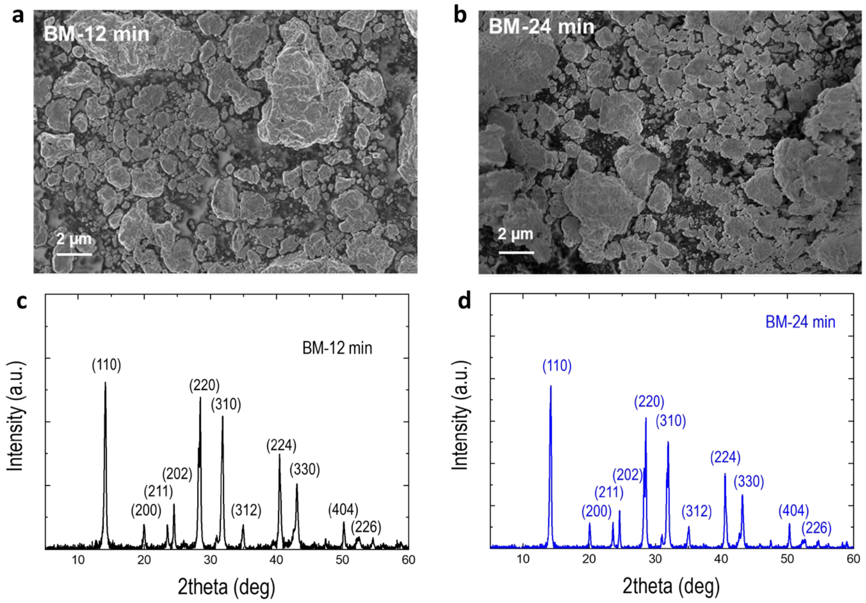

Supplementary Fig. 4 Characterizations of $MAPbI_3$ powders after ball milling (BM). SEM images of the BM as-synthesized powder samples of $MAPbI_3$ under different BM time of **(A)** 12 min and **(B)** 24 min, respectively. The corresponding XRD patterns of the BM as-synthesized powder samples of $MAPbI_3$ under different BM time of **(C)** 12 min and **(D)** 24 min, respectively. It should be noted that there is negligible difference between the BM samples under 12 min and 24 min. This is also supported by the XRD spectra where all the scattering peaks are assigned to the corresponding lattice plane of the $MAPbI_3$, and no impurity peaks of PbI_2 observed, which however occurs in certain solution-processed method in prior studies. As a result, we anticipate 12 min high energy BM process is enough to execute the complete reaction between the precursors.

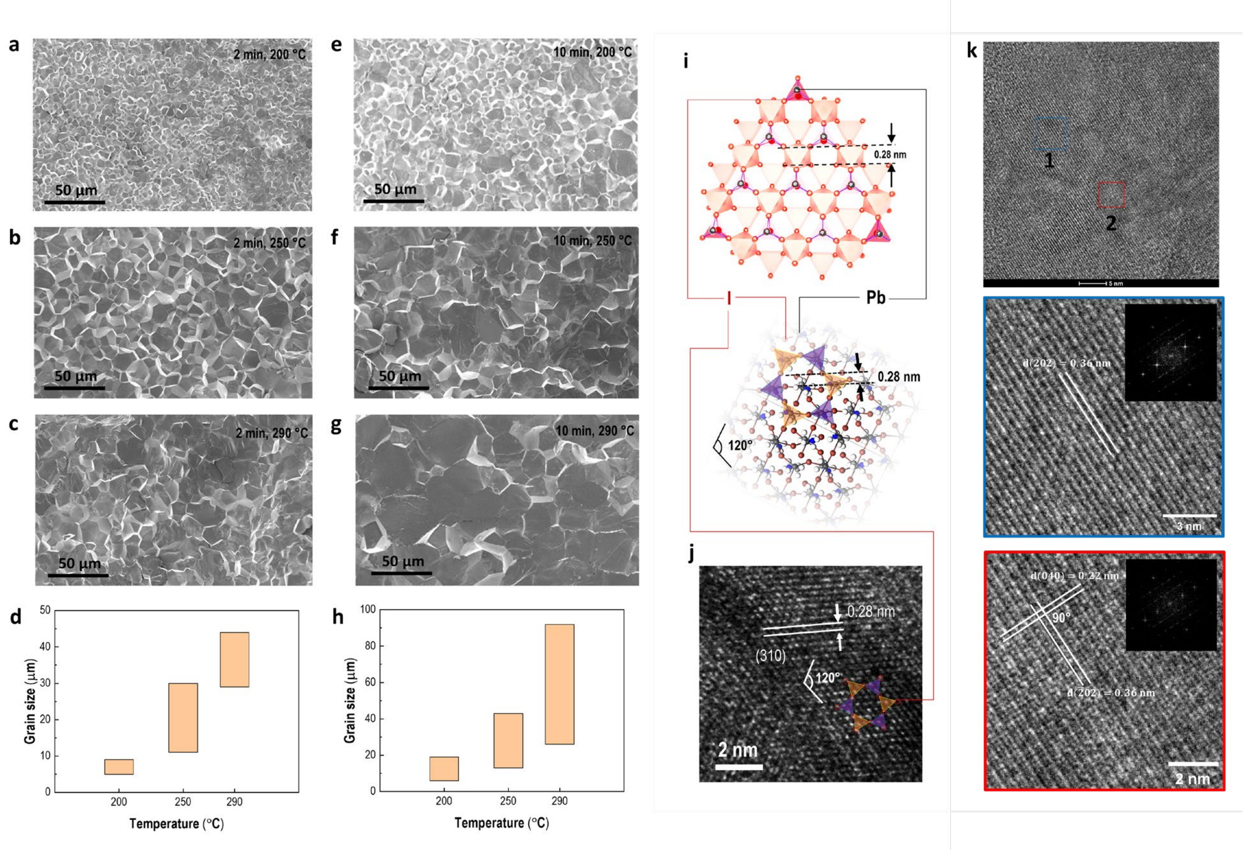

Supplementary Fig. 5 Microscopic features of FAST-$MAPbI_3$. (a-c) SEM images showing cross-sectional morphology of FAST-$MAPbI_3$ with processing temperature of **a** 200 °C, **b** 250 °C, and **c** 290 °C with processing time of 2 min. **d** Grain size statistics of the sample with processing time of 2 min. **(e-g)** SEM images showing cross-sectional morphology of FAST-$MAPbI_3$ with processing temperature of **e** 200 °C, **f** 250 °C, and **g** 290 °C with processing time of 10 min. **h** Grain size statistics of the sample with processing time of 10 min. These results show that by controlling the processing temperature and processing time, the grain size can be modulated in a significant range. Qualitatively, along with the time (from 2 to 10 min) and temperature (from 200 to 290 °C), the grain could monotonously grow from sub-10 μm to sub-100 μm. This can be understood by the necking growth theory[15]. **(i-k)** TEM results on FAST-$MAPbI_3$ sample: **i** schematic and atomic arrangement of a pseudo-cubic $MAPbI_3$ lattice. Heavy atoms of I and Pb are noted in the figure with a d-spacing of 0.28 nm of (310) and an interplanar angle of 120°. **j** an TEM image of the FAST-$MAPbI_3$ sample with the same perspective to that in **(i)**, showing identical values of d-spacing and interplanar angle to the molecular model in **(i)**. **k** TEM image of other locations in the same sample. Inset: the corresponding fast Fourier transform (FFT) image. We take two positions of "1" (blue) and "2" (red), and obtain the d-spacing of (040) and ($\bar{2}02$) planes with values of 0.22 and 0.36 nm, respectively. This is in consistent to the crystal feature of the $MAPbI_3$.

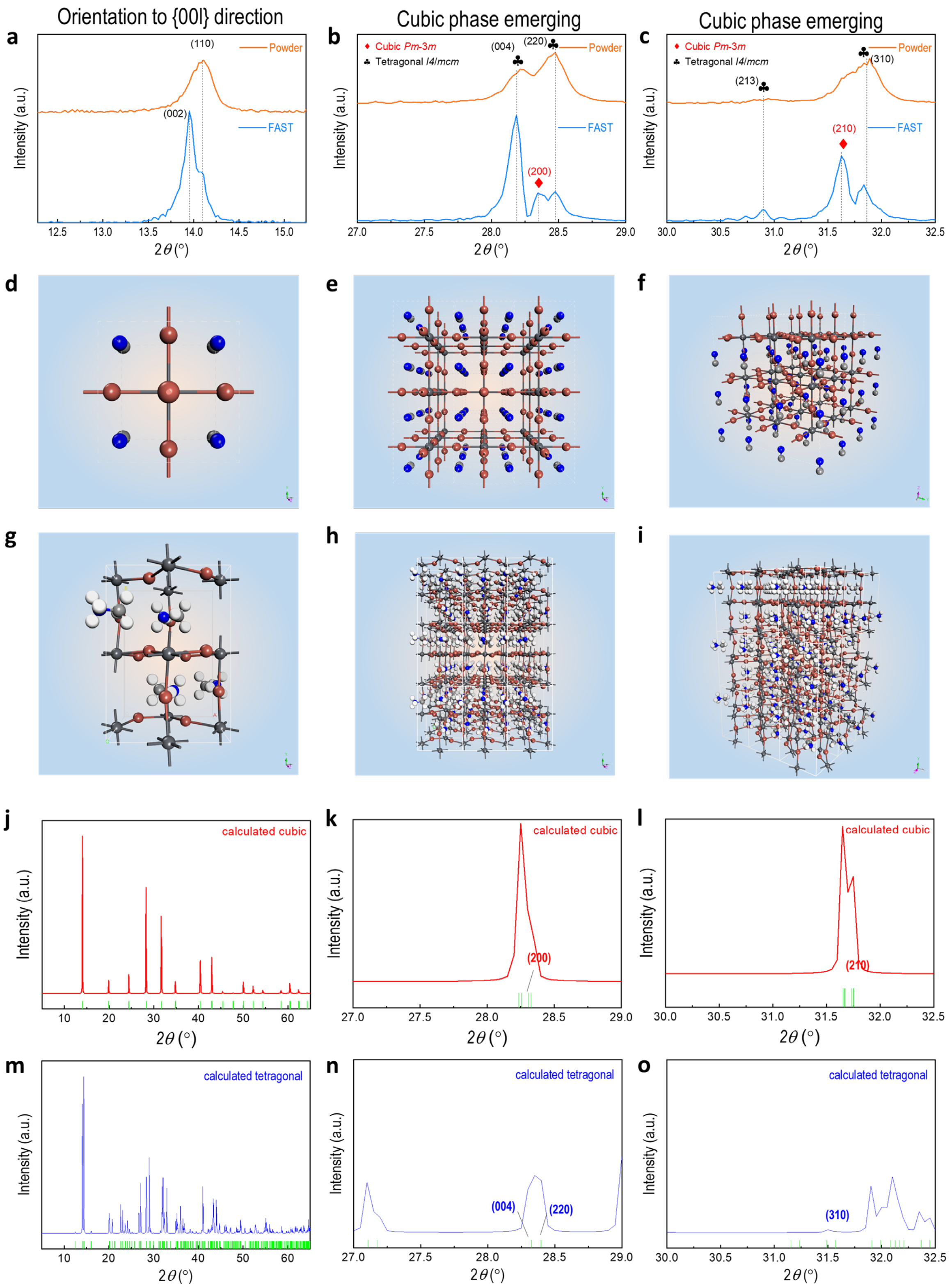

Supplementary Fig. 6 X-ray diffraction spectroscopy study on FAST-MAPbI₃. **a** Enlarged XRD spectra distinguishing the (110) and (002) scattering planes for powder and FAST sample. The FAST samples display the emergence of (00l) preferred orientation, compared to the powder

sample. (**b, c)** Enlarged XRD spectra showing the emerging cubic phase in the FAST sample, as marked by the red diamond in the figure. **d** Lattice configuration of unit cell of cubic $MAPbI_3$ with space group of $Pm\overline{3}m$, and its **e** 3×3×3-unit cell configuration projected to X-Y plane and **f** an arbitrary plane. **g** Lattice configuration of unit cell of tetragonal $MAPbI_3$ with space group of *I4/mcm*, and its **h** 3×3×3-unit cell configuration projected to X-Y plane and **i** an arbitrary plane. **j** Calculated XRD spectra of cubic $MAPbI_3$ with localized enlarged region for **k** (200) and **l** (210) planes. **m** Calculated XRD spectra tetragonal $MAPbI_3$ with localized enlarged region for **n** (004) & (220) and **o** (310) planes.

We compared the XRD spectra of FAST and powder $MAPbI_3$, with calculated XRD patterns from lattice of cubic and tetragonal models. The results show that the FAST-sample exhibits the emergence of (00l) dominated planes compared to the powders. In addition, evidence of the emerging planes of (200) and (210) of cubic phase support the presence of cubic phase $MAPbI_3$ in the sample. We chose the (200) and (210) planes of cubic phase to distinguish them from tetragonal phase is because they do not overlap with scattering planes of tetragonal phase. This can be seen from the calculated results of **Figs. S6j- Figs. S6o**. The cubic phase exhibits a well stretched Pb-I bonding with much less distortion compared to tetragonal phase. The presence of this cubic phase might be due to the high energy environment of FAST that endows sufficient energy for lattice relaxation and form the thermodynamically more stable cubic phase during the synthesis.

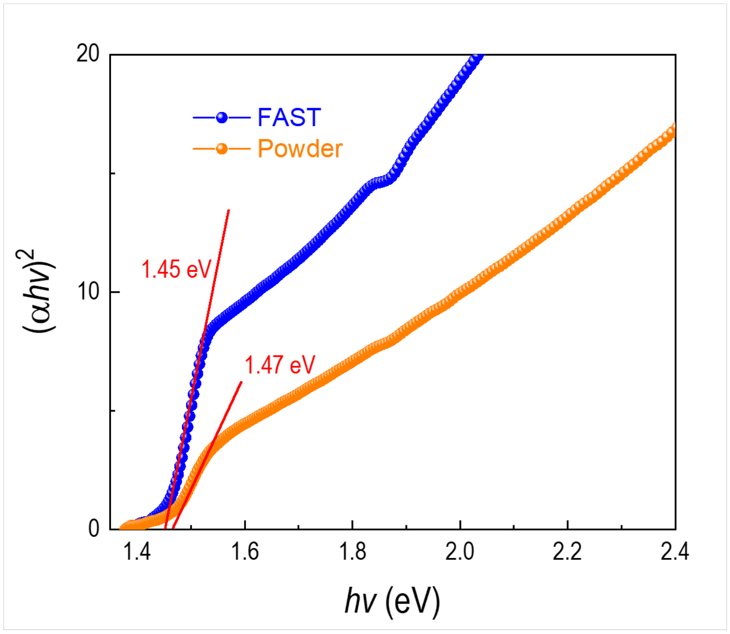

Supplementary Fig. 7 Tauc plot for powder and FAST-MAPbI$_3$. The optical band gap for both samples are determined to 1.46 eV. The Tauc plot reveals the absorption related quantity $(\alpha h\nu)^{\frac{1}{r}}$ with dependence of photo energy $(h\nu)$, with α being the absorption coefficient, h being the Planck's constant, and ν being the frequency of the light wave. According to Jan Tauc, the extrapolation of the linear region to the abscissa gives the energy of the optical bandgap of the material of interest. Depending on different type of materials, the order index r needs to be adjusted accordingly. In this study, we obtained the Tauc plot from the UV-vis absorption spectra. The MAPbI$_3$ displays a direct band gap feature[16], which assigns the r to be 1/2. As a result, we calculated the optical bandgap of the FAST-MAPbI$_3$ to be 1.46 eV. This value is smaller than the typical number of thin film samples (e.g., ~1.6 eV)[17], and closer to that of single crystals (e.g., 1.51 eV)[18]. Prior study proposed an indirect transfer mechanism to explain the smaller bandgap in thicker perovskite samples[19]. This can also be due to other effects such as spin-orbital coupling that induces Rashba splitting, and/or lattice distortion/phonon related effects that induced additional states close to the band edge[16].

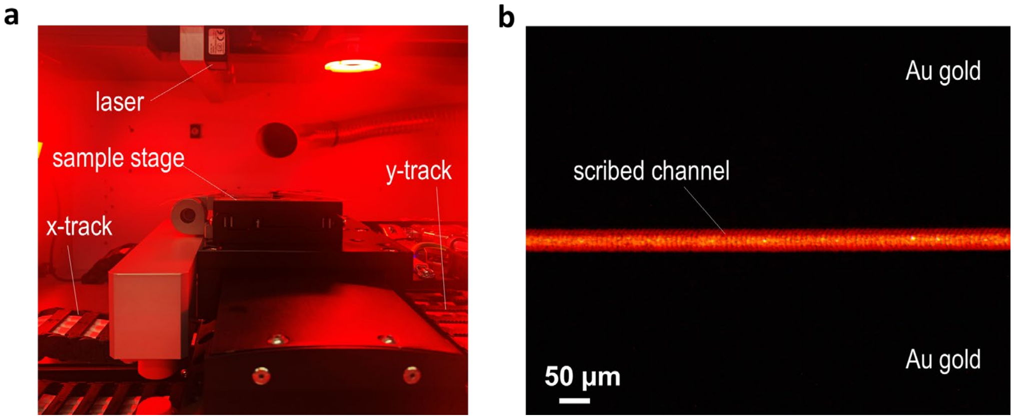

Supplementary Fig. 8 Manufacturing techniques for making FAST-$MAPbI_3$ photodetector. **(A)** Laser scribing technique photograph with key components presented. We utilized a pico-second laser to minimize the thermal damage to the sample. The laser power, scan rate, multi-scan times, z-focus, etc., are carefully controlled to peel off the Au layer without damaging the sample underneath. X & y-tracks are used to move the sample stage. The laser path is pre-programed through the integrated software from the machine. We also controlled the laser spot size by optical focusing system and optical path system, which resulted a small channel length to 36 µm. **(B)** Optical microscopic image showing the scribed channel which separate neighboring source and drain gold electrode.

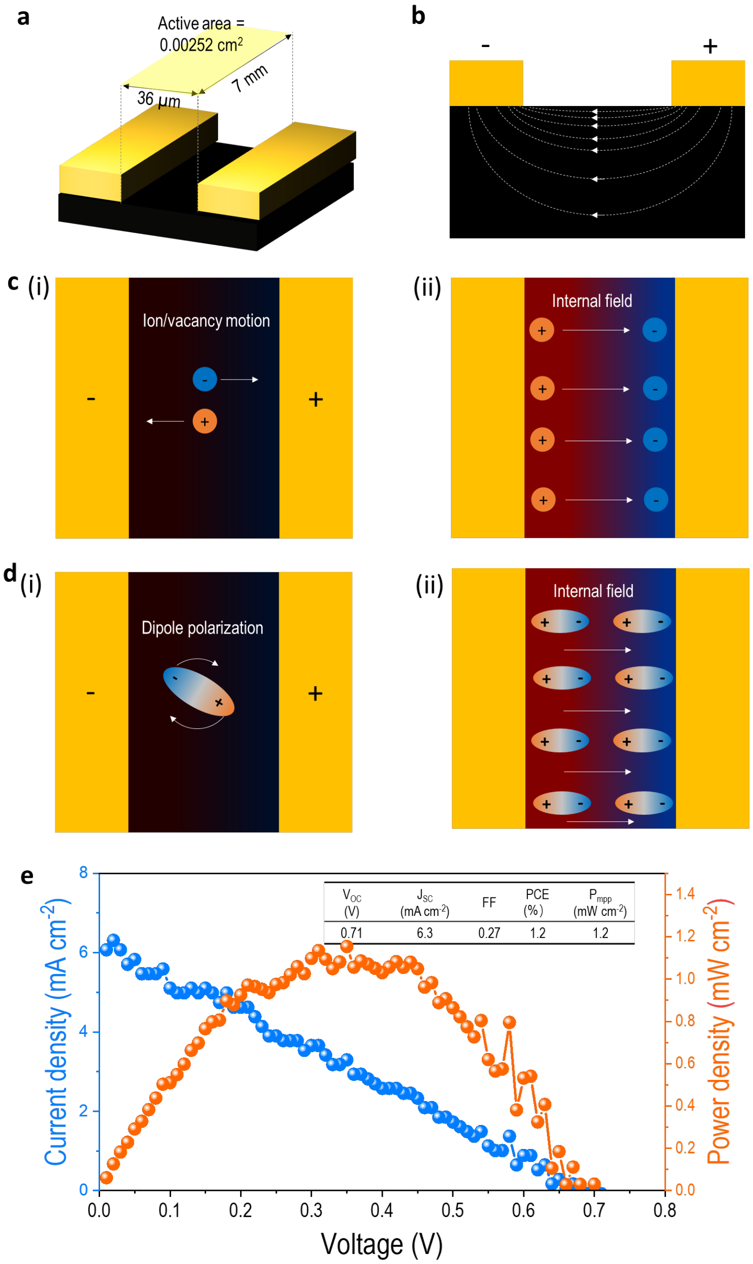

Supplementary Fig. 9 Solar cell performance of the FAST-MIM device. a Scheme of the FAST MIM device configuration with an active area of 0.00252 cm^2. **b** The electrical field distribution scheme at the cross-section of the MIM device, during the electric poling process. **c** Hypothetical mechanism of ion/vacancy motion induced electric asymmetry in MIM device: **(i)** ion drifting under poling bias and **(ii)** internal field formed due to the separated ions after poling. **d** Hypothetical mechanism of dipole polarization induced electric asymmetry in MIM device: **(i)** dipole reorientation under poling bias and **(ii)** internal field formed due to the polarized dipole after poling. **e** Current density-voltage curve of the MIM device after poling, with its corresponding power density-voltage curve. The solar cell performance parameters are also listed in the inserted table.

The lateral device can also be applied as a solar cell. Although the original device has a symmetric MIM configuration, it is possible to induce an internal electric asymmetric (internal field) by hypothetical mechanisms of ionic motion[20] (**Supplementary Fig. 9c**) and/or ferroelectric dipole polarization[21] (**Supplementary Fig. 9d**). As shown in **Supplementary Fig. 9c**, ion/vacancy within the lattice of the perovskites can be driven to move to the corresponding cathodes and anodes under the poling bias. After removal of the external poling bias, ions accumulated at corresponding electrodes can trigger the presence of an internal field which breaks the electric symmetry of the device. Similarly, the poling field can also induce the polarization of the electric dipole (either localized lattice distortion induced dipole or point defect induced dipole or other mechanisms) within the perovskites, which can also lead to the electric asymmetry of the device. Either of both mechanisms can lead to the internal electrical field that can drive the opposite motion of photo carriers. Accordingly, we measured the solar cell performance after poling the device under 8 V bias. The results show a solar cell power conversion efficiency (PCE) of 1.2%, with V_{OC} of 0.71 V, J_{SC} of 6.3 mA cm^{-2}, and FF of 27%. The relatively low performance is due to the lacking of proper buffer layers such as electron transfer layer (ETL) or hole transfer layer (HTL). Particularly the low FF of 27% is due to the poor selective extraction at the perovskite/gold interface that lacks sufficient barrier to block the reverse carrier transfer. Nevertheless, we found even using this simplest MIM device structure, the FAST device performance is still comparable with that of single-crystalline lateral device using C_{60}/BCP buffers[22] (PCE of ~2% without interfacial optimization).

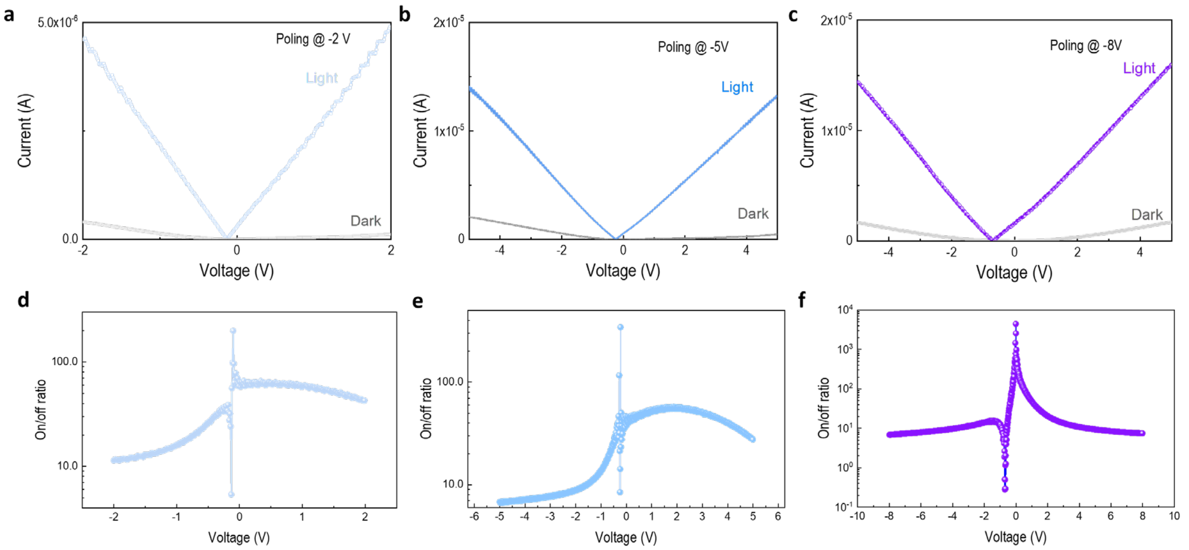

Supplementary Fig. 10 I-V curve and on/off ratio of MIM FAST-$MAPbI_3$ photodetectors. I-V curves of MIM photodetector under light and dark after poling with bias of **(a)** -2 V, **(b)** -5 V, and **(c)** -8 V. Corresponding on/off ratio at different bias after poling with bias of **(d)** -2 V, **(e)** -5 V, and **(f)** -8 V. The poling process can significantly induce an asymmetric internal field. For example, it can be seen from **Supplementary Fig. 10c**, after poling by -8 V, the absolute current value shows a minimal at -0.7 V. This is due to the bias induced polarization of the perovskite materials which can be the result of either ionic motion, or ferroelectric polarization, or other effects[23].

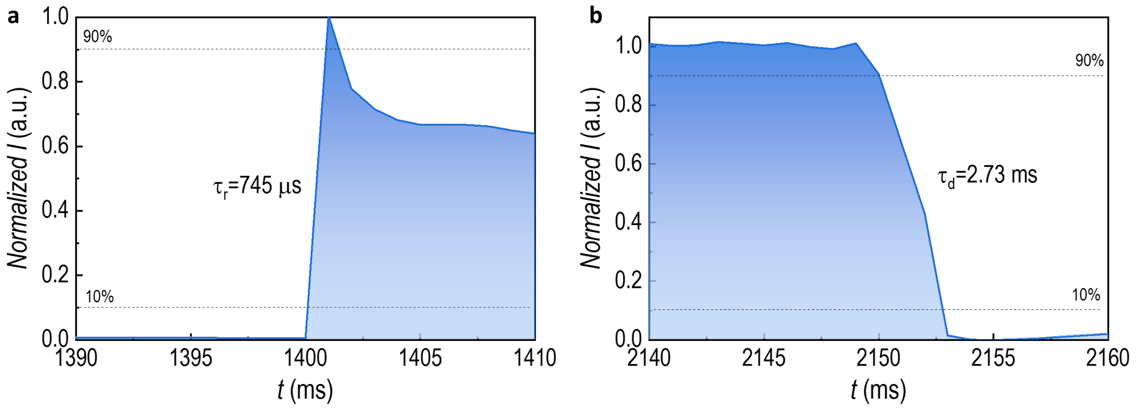

Supplementary Fig. 11 Normalized transient photocurrent of FAST-MAPbI$_3$ photodetector. **a** Raising part. **b** Dropping part.

The speed of photoresponse is a crucial figure-of-merit of photodetectors, which is determined by the transfer/extraction/collection rate of the photo-carriers. There are two parameters, i.e., raising time (t_r) and dropping time (t_d), where the former can be expressed by the equation of $t_r = \frac{L^2}{V\mu_n}$, with L is the inter-electrode spacing, V is the applied voltage, μ_n is the charge carrier mobility. The dropping time is related to the lifetime of the charge carriers within the device. We obtained both the raising time (t_r) and dropping time (t_d) from the normalized transient photocurrent of the FAST-MAPbI$_3$ photodetector device, by using the current values variant from 10% to 90% of its maximal number (**Supplementary Fig. 11**). As a result, raising time (t_r) and dropping time (t_d) of 745 μs and 2.73 ms have been obtained, respectively, suggesting a quick photoresponse, significantly more rapid than those of traditional In$_2$Se$_3$ and several ZnO-based photodetectors[24,25]. We also compared these characteristic times of our FAST-device with those of state-of-the-art solution-processed perovskite devices from prior reports (**Supplementary Table 2**). As can be seen in **Supplementary Table 2**, the raising time of the FAST-device is comparable to those of single crystalline perovskite devices, suggesting a fast transport behavior of the charge carriers in the FAST sample. To further reduce the response time, one possible solution is to shrink the active layer thickness. Since FAST-MAPbI$_3$ has a large thickness of 1.33 mm (compared to 100s nm scale of typical state-of-the-art perovskite photodetectors) but still exhibits small raising time, which allows sufficient space (e.g., reducing the thickness, or adding charge selective layers to accelerate the interfacial extraction, etc.) to further reduce the response time of the device.

On the other hand, from the raising time, the charge carrier mobility can also be estimated. The calculated mobility from this transient raising method is 44.9 cm^2 V^{-1} s^{-1}, which is one order of magnitude higher than the number estimated from the SCLC method. This discrepancy might be ascribed to different measuring methods that require certain boundary conditions or equation application precondition of the model that can be slightly deviated from the real device. Nevertheless, regardless of the measuring methods, the mobility number of the SCLC-MAPbI$_3$ is still in the range of the single crystal.

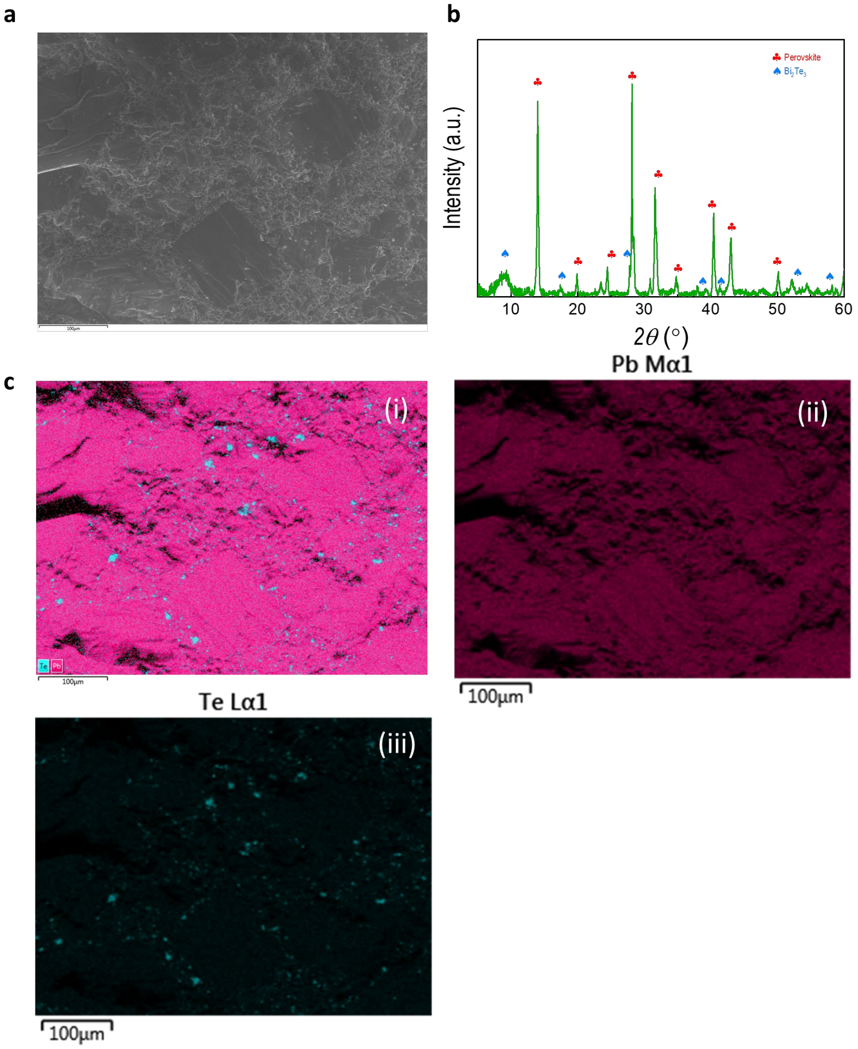

Supplementary Fig. 12 Characterizations of $(Bi_2Te_3)_{0.1}(MAPbI_3)_{0.9}$ alloy. a SEM image showing the perovskite grain being surrounded by the Bi_2Te_3, **b** XRD pattern displaying both sets of scattering planes of perovskite and Bi_2Te_3, and **c** a corresponding EDS mapping of $(Bi_2Te_3)_{0.1}(MAPbI_3)_{0.9}$: **(i)** element mapping of both Pb and Te in the alloy. Separate element mapping of **(ii)** Pb and **(iii)** Te (EDS peaks of Bi and Pb locate closely with each other and difficult to distinguish Bi from Pb). From the **Supplementary Fig. 12c**, the alloy shows a well dispersion of perovskite particles in the matrix of Bi_2Te_3, with the size of hundreds of nanometers to ca. 7 μm. From the individual element mapping, for example of Pb (**Supplementary Fig. 12c (ii)**), there is a continuous map of Pb covering the whole SEM image area, suggesting a well dispersion of perovskite over the whole sample. Overall, the results show the dispersion of perovskite in the Bi_2Te_3 is uniform and no impurities or interference induced new phased emerged (as evidenced by the XRD in **Supplementary Fig. 12b**).

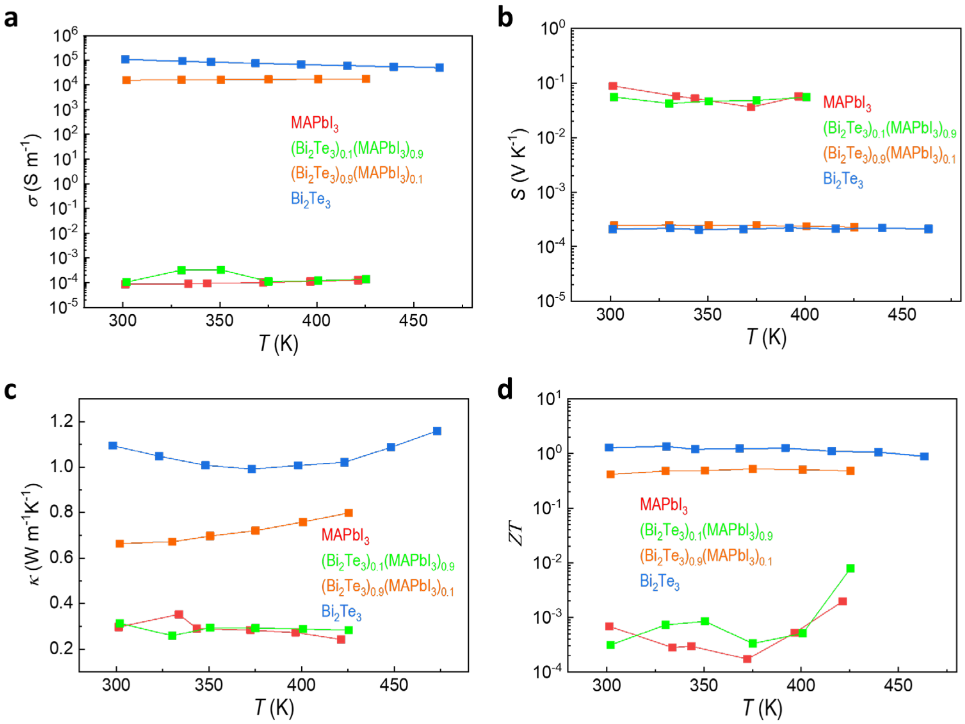

Supplementary Fig. 13 Thermoelectric properties of the FAST-$(Bi_2Te_3)_x(MAPbI_3)_{1-x}$ alloys. **a** Electrical conductivity (σ), **b** Seebeck coefficient (S), **c** thermal conductivity (κ), and **d** ZT value. In general, compared to bismuth telluride which is metallic material with high electrical and thermal conductivity but low Seebeck coefficient, halide perovskites are more insulative with low electrical and thermal conductivity but high Seebeck coefficient. However, the magnitude of electrical conductivity is around 9 orders of magnitude but the compensation in Seebeck coefficient is around ca. 3 orders. Considering less than 1 order of magnitude lower thermal conductivity, the overall ZT ($zT = \frac{S^2\sigma}{\kappa}T$) of FAST-$MAPbI_3$ is still ca. 2-order of magnitude lower than bismuth telluride. We study the two extreme cases of $(Bi_2Te_3)_x(MAPbI_3)_{1-x}$, with x being 10% or 90%, to understand how the alloy feature can modulate the overall performance. As expected, all the alloy shows intermediate thermoelectric properties to their corresponding matrix only counterparts, suggesting no coupling effect.

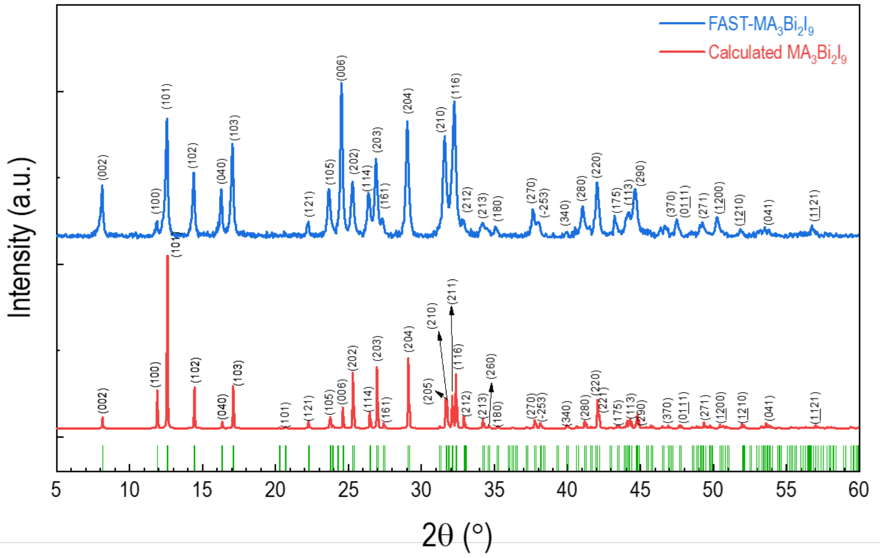

Supplementary Fig. 14 Experimental and calculated XPD spectra of FAST-$MA_3Bi_2I_9$. The calculated XRD is based on a standard lattice model obtained from an online open database for $MA_3Bi_2I_9$. All the peaks from the FAST sample are assigned to the corresponding lattice planes, suggesting no impurities present in the sample.

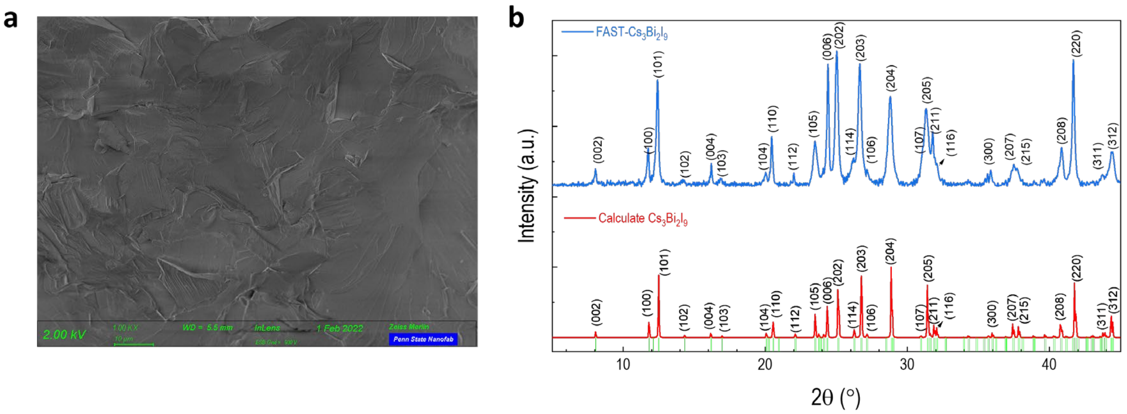

Supplementary Fig. 15 Supplementary characterizations of 2D all-inorganic FAST-$Cs_3Bi_2I_9$. a SEM image of the cross-section of the sample, **b** XRD pattern of the sample with comparison to the calculated results with noted corresponding scattering planes.

The SEM shows compact grain with barely noticeable grain boundaries. The grain exhibits large size over 10 μm. We also characterize the crystalline feature by XRD. The calculated XRD is based on a standard lattice model obtained from an online open database. We compared the results with corresponding scattering planes in **Supplementary Fig. 15b**. All the scattering peaks can be assigned to the corresponding lattice planes, without the presence of any impurity peaks. These results indicate the high quality of the FAST-$Cs_3Bi_2I_9$.

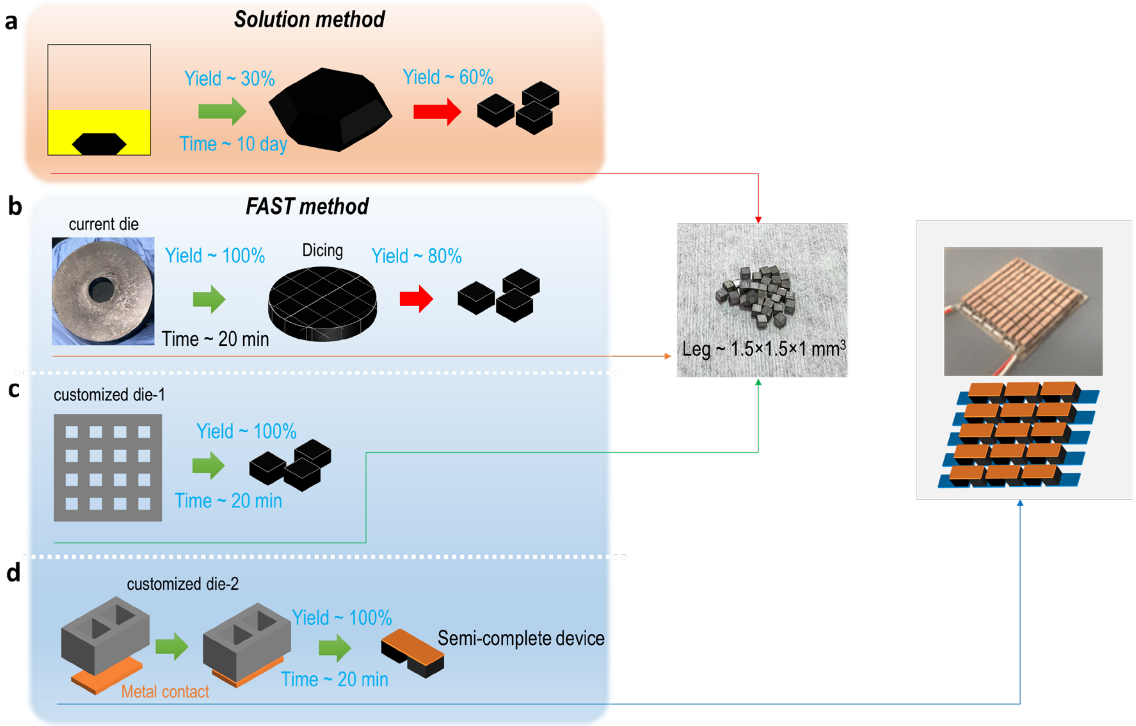

Supplementary Fig. 16 Proposed manufacturing of thermoelectric legs from FAST-perovskites. a Conventional solution method which is of low material usage (ca. 30% yield) and long processing time (weeks level for growing large size crystal), representing a less efficient way for manufacturing. **b** FAST methods with 100% synthetic yield to a disk sample, but still exhibit material losses (80% yield in mechanical dicing) during dicing due to fringe loss and also failure due to the fragile nature of the material. **c** Proposed 3D printing technique using FAST-based methods for halide perovskites. A customized die can be predesigned which can be used to mold the perovskite thermoelectric leg. The cubic legs can then be directly used for device assembly. **d** Proposed 3D printing for semi-finished device (an p-n unit), where two dies containing n-type and p-type perovskites (can be realized by proper doping strategy) connected with a metal and conductive binder substrate. After FAST, the device can be ready to assembly into a p-n unit, which can be further assembled into an array.

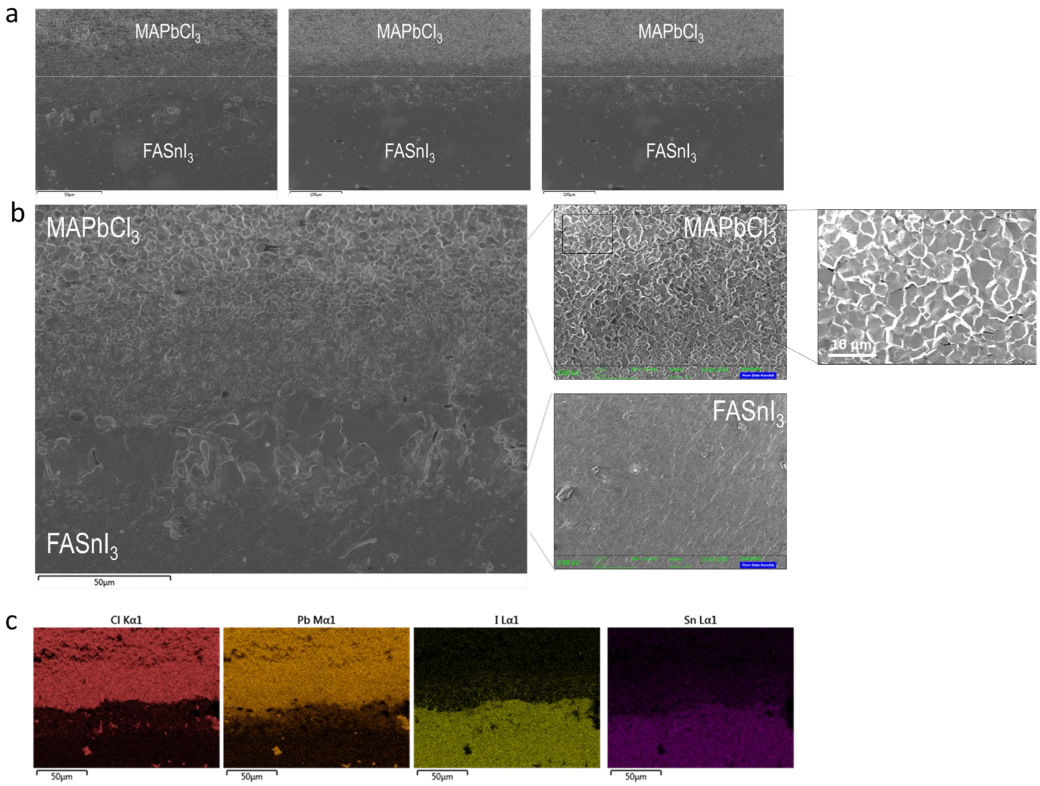

Supplementary Fig. 17 $MAPbCl_3$/$FASnI_3$ heterojunction. **a** cross-sectional SEM images showing multiple sites of the hetero-interface. **b** Magnified cross-sectional SEM at the hetero-interface, showing an intimate interface between different perovskites. **c** EDS mapping of different elements, indicating two perovskites closely jointing together at the interface.

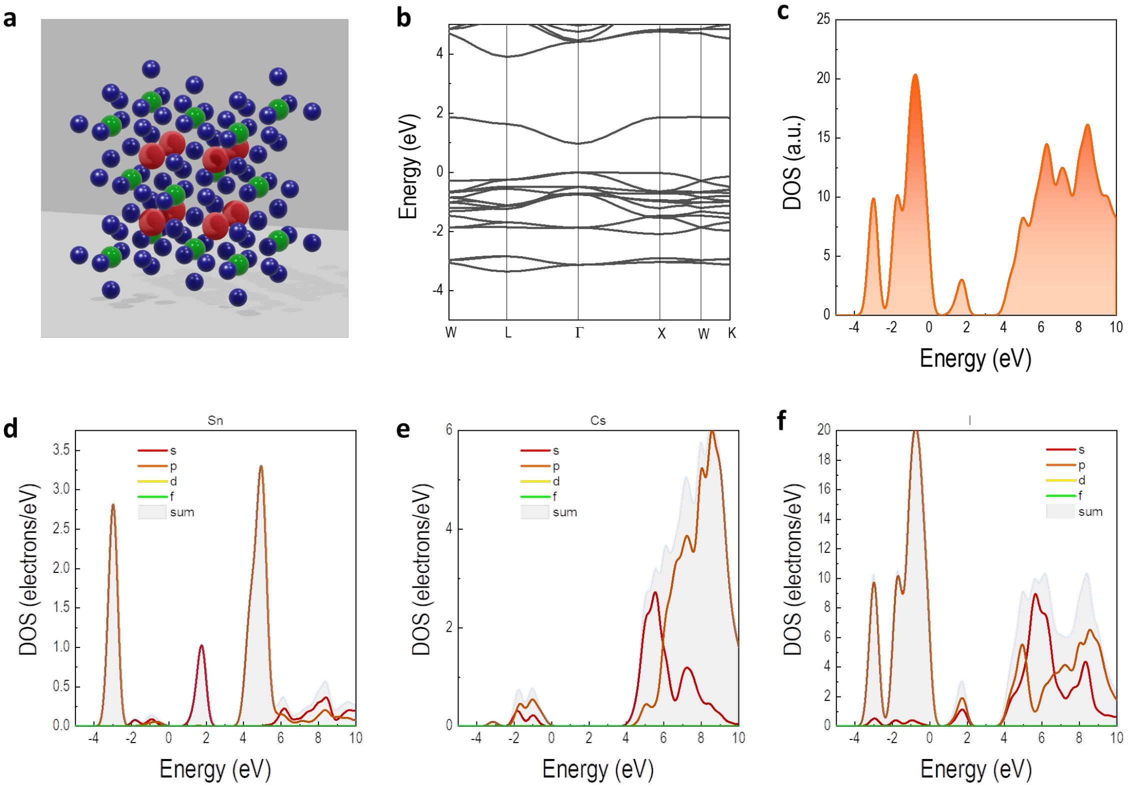

Supplementary Fig. 18 Calculated electronic band structure of the vacancy-ordered Cs_2SnI_6 perovskite. **a** Crystal lattice diagram of Cs_2SnI_6, where the green bead is the Sn, indigo bead is the I, and red bead is Cs, respectively. **b** Electronic band structure of Cs_2SnI_6, with a band gap of 1.25 eV which is consistent with prior reported experimental value of 1.26 eV[26]. **c** Corresponding total Density of State (DOS) of Cs_2SnI_6, with separate DOS of **(d)** Sn, **(e)** Cs, and **(f)** I, respectively, with their individual orbital contribution from s, p, d, f as well as their sum.

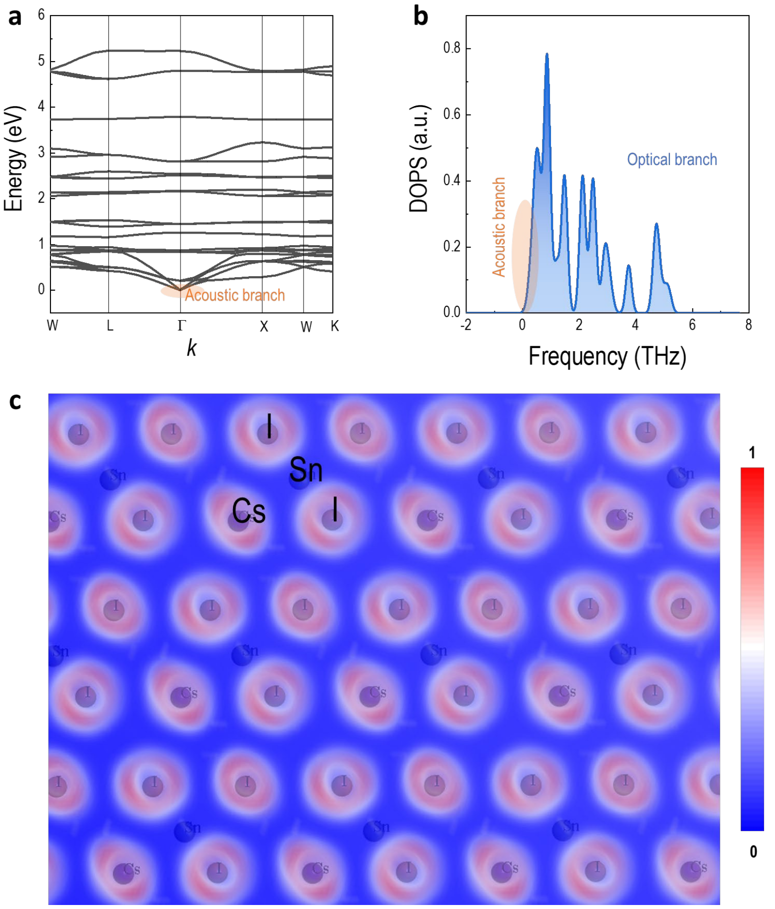

Supplementary Fig. 19 Calculated phonon related properties of the vacancy-ordered Cs_2SnI_6 perovskite. a Phonon band structure of Cs_2SnI_6 perovskite. **b** Density of phonon state (DOPS) of the Cs_2SnI_6, showing no unstable negative components which is consistent to its stable nature. **c** Normalized electron localization function (ELF) mapping, projected on the [110] plane. This spatial arrangement of the localized electron density surrounding each atom can visualize the degree of phonon anharmonicity[27] in the lattice and thereby influence the lattice thermal conductivity (κ_l). As can be seen from **Supplementary Fig. 19c**, the map shows the localized dimensionless ELF probability density, with normalization from 0 to 1. The higher electronegative element of I (electronegativity of 2.66) attracts more charge than Sn (1.96), resulting negligible electrons surrounding Sn and hence a more ionic-like bond in the lattice. More importantly, non-spherical electron density for both I and Cs are present, which explains the origin of the large phonon anharmonicity in the lattice. This will increase the phonon scattering and thereby suppress the lattice thermal conductivity (κ_l). Such a phonon anharmonicity is typically used to explain the low κ_l in other halide materials[28,29].

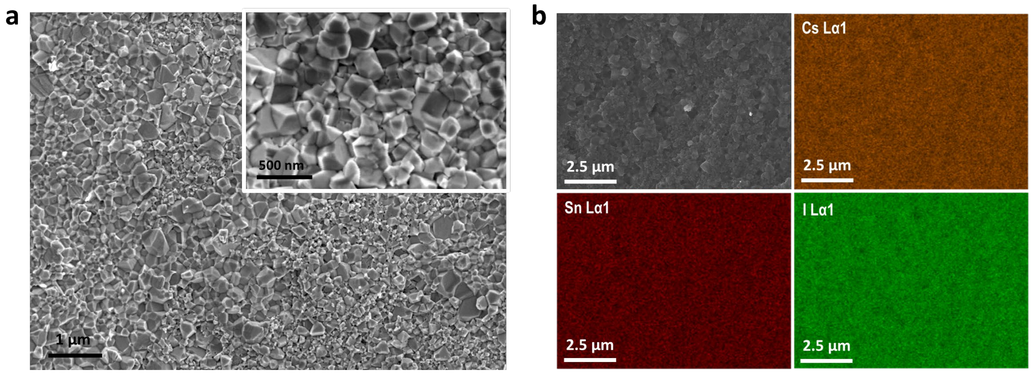

Supplementary Fig. 20 Morphology of FAST-Cs_2SnI_6. (A) SEM images showing the cross-section of the FAST-Cs_2SnI_6 sample with enlarged image displaying average grain size around 400 nm. **(B)** EDS images of the sample with element mapping of Cs, Sn, and I, indicating a uniform crystal phase of the sample.

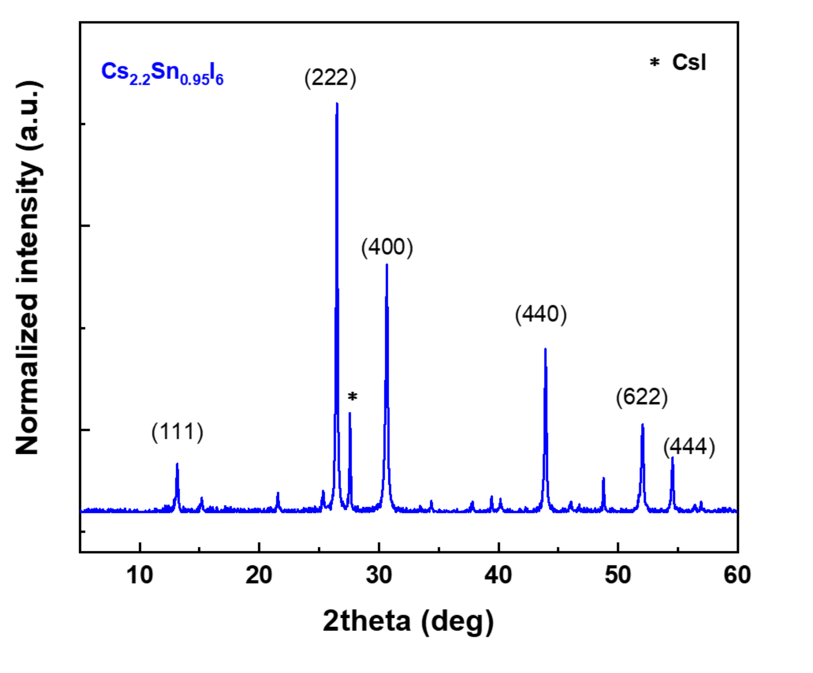

Supplementary Fig. 21 The XRD spectra of the FAST-$Cs_{2.2}Sn_{0.95}I_6$ perovskite. The impurity peak of CsI is at 2θ of 27.6 °.

As demonstrated in the main text, we found the stoichiometric Cs_2SnI_6-FAST sample still displays the impurity phase of CsI due to the fast nanostructure facilitated degradation during the BM sample preparation process. By reducing the CsI ratio in the precursors, the final FAST-$Cs_{1.9}Sn_{1.025}I_6$ sample can exhibit purer phase without the presence of CsI (**Fig. 4B(iii)**). On the contrary, by adding extra CsI, such CsI XRD peak intensity increases. By compiling the results of XRD of different samples, it is concluded that the SnI_4 is a sacrifice agent during the ball milling process. With about 5 mol% extra SnI_4, the final product can keep a purer phase with minimized CsI impurity.

Interestingly, by using a stoichiometric ratio of precursor powder of CsI and SnI_4, we observed an impurity XRD peak of CsI at 2θ of 27.58° (**Fig. 4B(iii)**). Notwithstanding the air-stable nature of Cs_2SnI_6, this CsI impurity phase has been observed in many solution methods due to a quick decomposition from Cs_2SnI_6 to CsI which can be ascribe to the formation of micro etch pits[30,31]. This perhaps occurs during the transfer process between ball milling process and the FAST synthesis, as the milled nano/meso powder offers more etch pits to decompose the material. To avoid so, we adjust the ratio of precursors before milling and execute an identical FAST process for all the samples. **Fig. 4B(iii)** also compares XRD spectra of FAST samples from the stoichiometric to 2.5 mol% extra SnI_4 and 5 mol% extra CsI. We found that addition of extra 2.5 mol% of SnI_4 could stabilize the crystal during the milling and eventually eliminate the CsI impurity. In comparison, samples with more CsI (10 mol% extra) in the precursor brings more CsI to the final product (**Supplementary Fig. 21**).

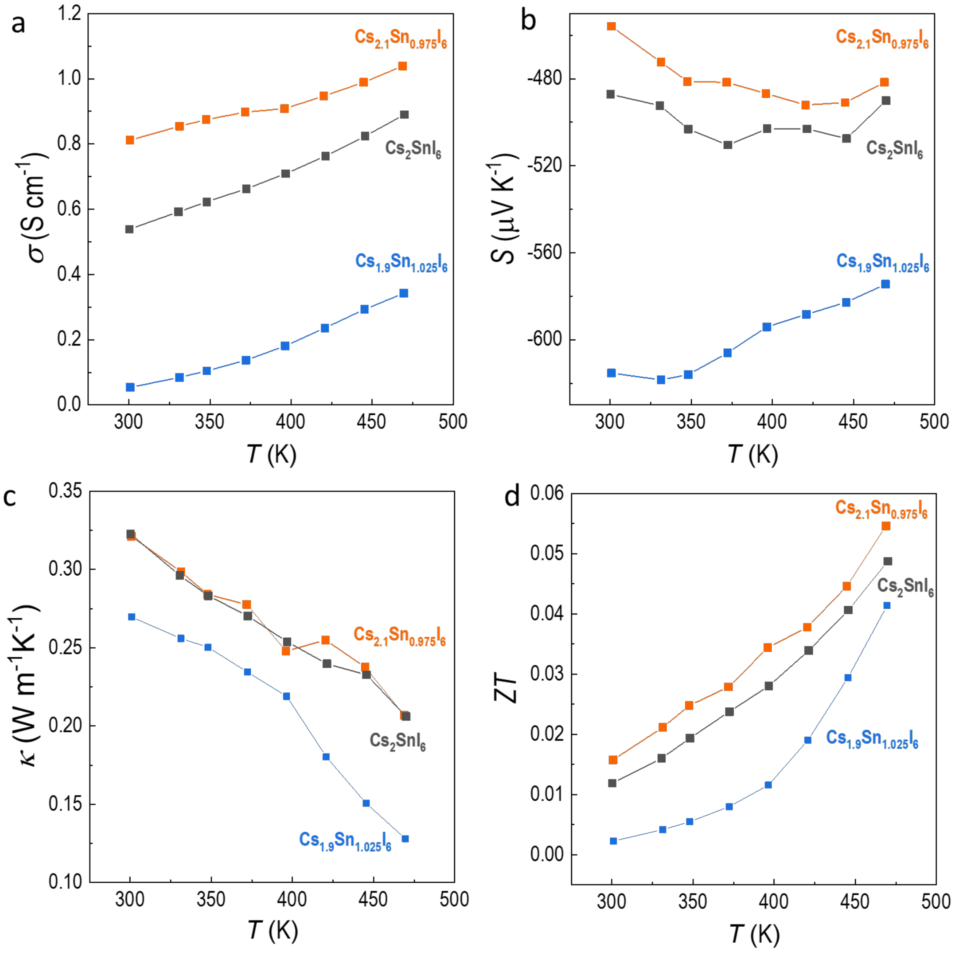

Supplementary Fig. 22 Thermoelectric properties of FAST-$Cs_{2.1}Sn_{0.975}I_6$, Cs_2SnI_6, and $Cs_{1.9}Sn_{1.025}I_6$, respectively. Temperature dependent **(a)** electrical conductivity (σ), **(b)** Seebeck coefficient (S), **(c)** thermal conductivity (κ), and **(d)** ZT value.

The FAST-$Cs_{1.9}Sn_{1.025}I_6$ with purer phase shows an inferior electrical conductivity of 0.05 S cm^{-1} at 300 K, which is consistent with the cold-pressed Cs_2SnI_6 after annealing (ca. 0.01 S cm^{-1}). Along with the addition of extra CsI, the electrical conductivity of the final FAST sample increases. The FAST-$Cs_{2.1}Sn_{0.975}I_6$ exhibits the highest electrical conductivity of 1.04 S cm^{-1} at 469 K. All the FAST samples show a negative Seebeck coefficient revealing the n-type nature of the material.

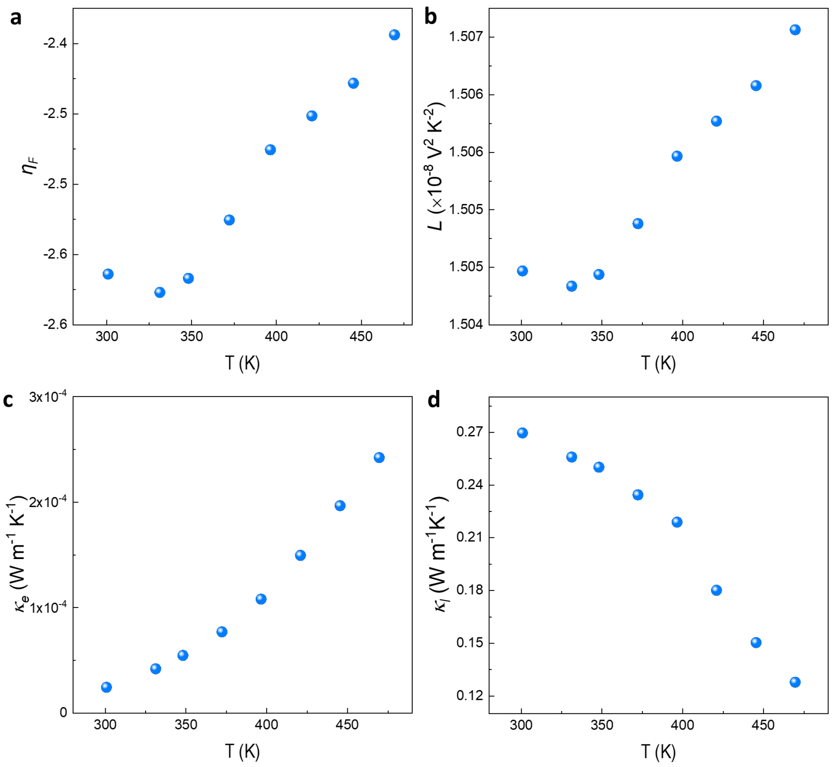

Supplementary Fig. 23 Temperature dependent properties. **(a)** Reduced Fermi level, **(b)** Lorenz factor, **(c)** electronic thermal conductivity, and **(d)** lattice thermal conductivity of FAST-$Cs_{1.9}Sn_{1.025}I_6$ perovskite.

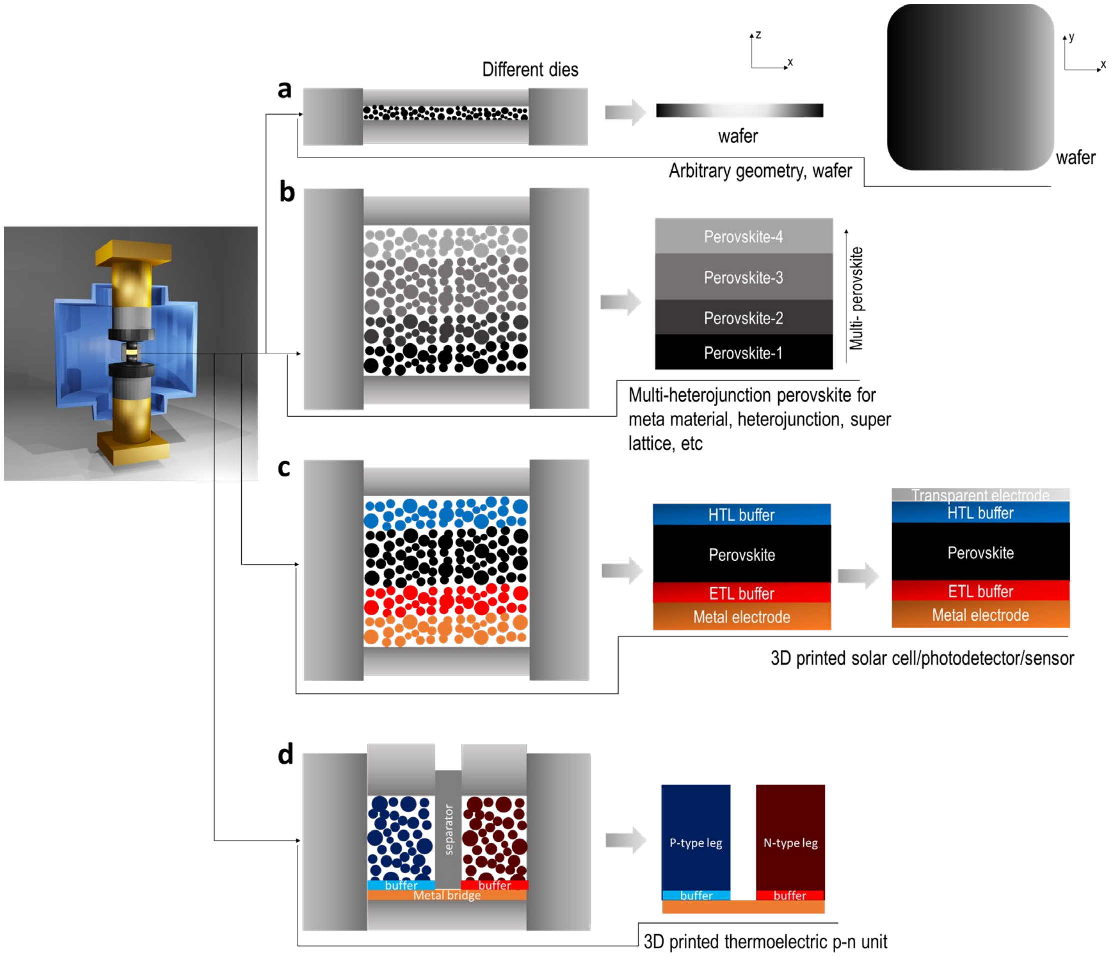

Supplementary Fig. 24 Derived FAST-incorporated techniques. a FAST uses dies of arbitrary geometries enabling directly fabrication of a perovskite wafer or a geometry of predesigned shape using specific die. **b** Manufacturing of gradient perovskites. **c** Direct printing a p-i-n junction, using different powders. **d** Direct printing of p-n thermoelectric leg.

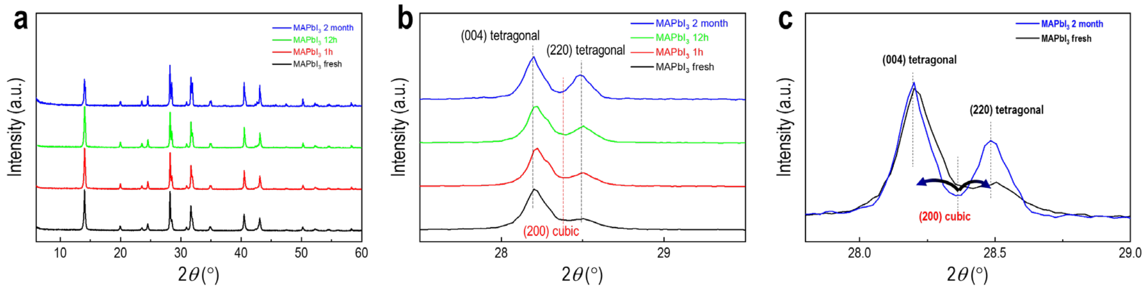

Supplementary Fig. 25 Stability study of the FAST $MAPbI_3$ samples. a XRD results of a EM-FAST $MAPbI_3$ pellet stored in the ambient atmosphere for different time (RH 35-80%, depending on daily whether at State College, PA, United State, temperature of ca. 25 °C controlled by lab). **b** Local magnification of the XRD results, showing the phase evolution from cubic (200) planes towards tetragonal (004) and (220) planes. c Comparison of fresh and 2-month age XRD, displaying the phase evolution. These results suggest robust feature of the EM-FAST $MAPbI_3$ samples (no obvious degradation even after ambient storage for 2 months). The origin may come from the hypothetical lattice strain in these samples, which could increase the activation energy of ion migration[32,33].

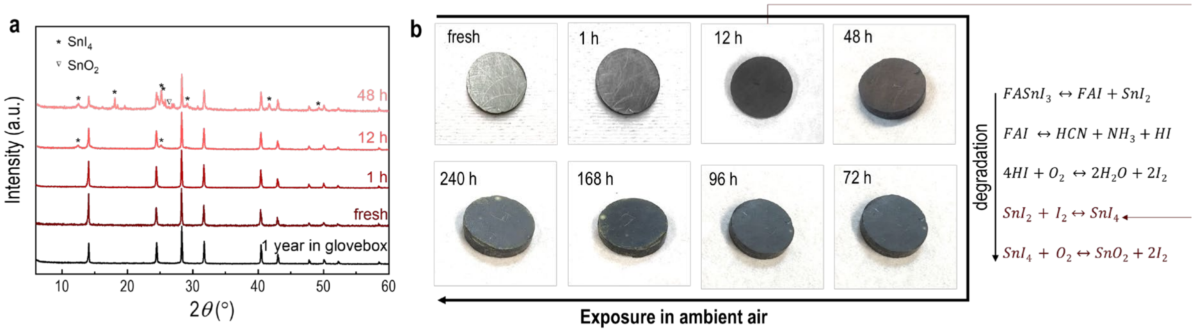

Supplementary Fig. 26 Stability study of the FAST $FASnI_3$ samples. a XRD results of a EM-FAST $FASnI_3$ pellet stored in the ambient atmosphere for different time (RH 35-80%, depending on daily whether at State College, PA, United State, temperature of ca. 25 °C controlled by the lab). **b** Photography of the $FASnI_3$ sample with different ages from 0 h to 24 h, exposed in ambient air. A degradation chemical reaction set is inserted to show the compositional evolution.

First of all, it is shown that in the case of storage inside of a glovebox, even after 1 year, the sample does not show any impurities or degradation peaks in the XRD. While storing in the ambient air, after 12 h there is emergence of SnI_4 phase, and after 48 h there is emergence of SnO_2 phase. This evolution is consistent to the sequential degradation process in the equation set in **Supplementary Fig. 26b**. While from the photo, it is hard to observe obvious color change of the sample. This observation, together with the remaining scattering peaks of $FASnI_3$ in the 48-h age sample in **Supplementary Fig. 26a**, suggest there could be a protection layer formed by the degrades covering the surface, which can prevent further degradation of the inner sample.

Supplementary Tables

Supplementary Table 1 Parameters of TRPL measurement for thin film, powder, and FAST-$MAPbI_3$.

Sample	A_1	τ_1 (ns)	A_2	τ_2 (ns)	τ_{ave} (ns)
Thin film-$MAPbI_3$	0.57	30.2	0.43	77	61.1
Powder-$MAPbI_3$	0.63	6.7	0.37	63	54.3
Single crystal-$MAPbI_3$	0.18	1.4	0.82	25	20.7
FAST-$MAPbI_3$	0.56	72.4	0.44	311	257.1

Supplementary Table 2 Raising time of $MAPbI_3$ based photodetectors.

Materials	Synthetic method	Crystal nature	Raising time	Falling time	Reference
$MAPbI_3$	solution	single crystal	<0.2 s	<0.2 s	[34]
$MAPb(Br_{0.78}I_{0.22})_3$	solution	single crystal	3.4 ms	3.6 ms	[35]
$MAPbI_3$	solution	single crystal	71 µs	113 µs	[36]
$MAPbI_3$	solution	thin film	<40 ms	<50 ms	[37]
FAST-$MAPbI_3$	FAST	polycrystal	800 µs	3.33 ms	This work

Supplementary Table 3 Comparison of Seebeck coefficient for different materials.

Materials	Temperature (K)	S (μV K^{-1})	Reference
$MAPbI_3$ single crystal	300-330	920	38
	351	1693	
$MAPbI_3$ thin film	293-373	-6500	39
		5500	
$MAPbI_3$ single crystal	295	820	40
$MAPbI_3$ thin film	330	-17000 (under light)	41
$MAPbI_3$ polycrystal	300	-1350	42
Bi_2Te_3	300	208	43
$Mg_3Bi_{1.25}Sb_{0.75}$	315	-220	44
Selenium	333	900	45
FAST-$MAPbI_3$	300	90000	This work

Supplementary Table 4 Comparison of thermal conductivity for different $MAPbI_3$.

$MAPbI_3$	Temperature (K)	κ (Wm^{-1} K^{-1})	Reference
Single crystal	300	0.5	46
polycrystal	300	0.3	
Single crystal	298-425	0.3-0.42	38
Thin film	298	0.33	38
Single crystal	298	0.34	47
Single crystal	298	0.3	48
FAST-$MAPbI_3$	300	0.3	This work

Supplementary Table 5 Summary of FAST-synthesized perovskites in this work.

Materials	Composition	Precursors	FAST	Product	Density
**Prototype**	$MAPbI_3$	MAI & PbI_2	52 MPa; 200 °C	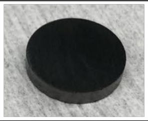	4.16 g cm^{-3}
**Alloy**	$(Bi_2Te_3)_{0.1}(MAPbI_3)_{0.9}$	$MAPbI_3$ & Bi_2Te_3	52 MPa; 250 °C	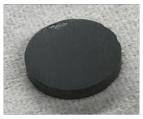	4.24 g cm^{-3}
	$(Bi_2Te_3)_{0.9}(MAPbI_3)_{0.1}$			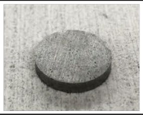	6.36 g cm^{-3}
**heterojunction**	$MAPbI_3$/conductive polymers	$MAPbI_3$ & conductive compound (ProbeMet^{TM})	29 MPa; 150 °C	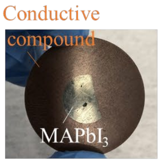	/
	$MAPbCl_3$/$FASnI_3$	MACl & $PbCl_2$ /FAI & SnI_2	52 MPa; 200 °C	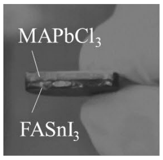	/
**Lead-free**	$MASnI_3$	MAI & SnI_2	52 MPa; 150 °C	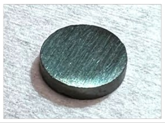	/
	$CsSnI_3$	CsI & SnI_2	52 MPa; 300 °C	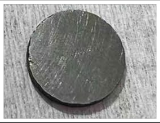	4.59 g cm^{-3}
	$FASnI_3$	FAI & SnI_2	52 MPa; 150 °C	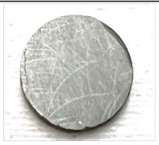	/
**2D**	$(MA)_3Bi_2I_9$	MAI & BiI_3	52 MPa; 300 °C	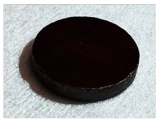	4.10 g cm^{-3}
	$MA_2CuCl_2Br_2$	MACl & $CuBr_2$	52 MPa; 120 °C	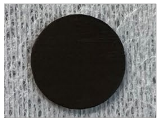	/

**All-inorganic**	$Cs_3Bi_2I_9$	CsI & BiI_3	52 MPa; 500 °C	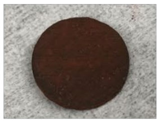	4.62 g cm^{-3}
	Cs_2SnI_6	CsI & SnI_4	52 MPa; 300 °C	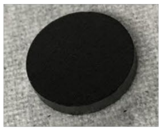	4.73 g cm^{-3}
**Color (larger bandgap)**	$MAPbCl_3$	MACl & $PbCl_2$	52 MPa; 200 °C	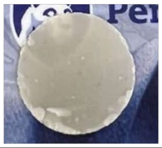	/
	$FAPbBr_3$	FABr & $PbBr_2$	52 MPa; 180 °C	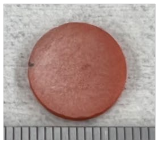	/

Supplementary References

1. Freitas, A. A., MaçAnita, A. A. L. & Quina, F. H. Improved analysis of excited state proton transfer kinetics by the combination of standard and convolution methods. *Photochem. Photobiol. Sci. 2013 125* **12**, 902–910 (2013).
2. Santra, K. *et al.* What is the best method to fit time-resolved data? A comparison of the residual minimization and the maximum likelihood techniques as applied to experimental time-correlated, single-photon counting data. *J. Phys. Chem. B, Condens. Matter, Mater. Surfaces, Interfaces Biophys. Chem.* **120**, 2484–2490 (2016).
3. Hou, Y. *et al.* Enhanced Performance and Stability in DNA-Perovskite Heterostructure-Based Solar Cells. *ACS Energy Lett.* **4**, 2646–2655 (2019).
4. Sillen, A. & Engelborghs, Y. The Correct Use of "Average" Fluorescence Parameters. *Photochem. Photobiol.* **67**, 475–486 (1998).
5. Langmuir, I. The Effect of Space Charge and Residual Gases on Thermionic Currents in High Vacuum. *Phys. Rev.* **2**, 450 (1913).
6. Lampert, M. A. Simplified Theory of Space-Charge-Limited Currents in an Insulator with Traps. *Phys. Rev.* **103**, 1648 (1956).
7. Murgatroyd, P. N. Theory of space-charge-limited current enhanced by Frenkel effect. *J. Phys. D. Appl. Phys.* **3**, 151 (1970).
8. Mott, N. & Gurney, R. *Electronic processes in ionic crystals*. (Oxford, Clarendon Press, 1940).
9. Zhu, W. *et al.* Tunable optical properties and stability of lead free all inorganic perovskites ($Cs_2SnI_xCl_{6-x}$). *J. Mater. Chem. A* **6**, 2577–2584 (2018).
10. Zhu, W. *et al.* Deciphering the degradation mechanism of the lead-free all inorganic perovskite Cs2SnI6. *npj Mater. Degrad. 2019 31* **3**, 1–7 (2019).
11. Snyder, G. J. & Toberer, E. S. Complex thermoelectric materials. *Nat. Mater. 2008 72* **7**, 105–114 (2008).
12. Jones, W. & March, N. H. (Norman H. *Theoretical Solid State Physics, Vol. 2: Non-Equilibrium and Disorder (Non-Equilibrium & Disorder)*. (Dover Publications, 1985).
13. Bahk, J. H., Bian, Z. & Shakouri, A. Electron energy filtering by a nonplanar potential to enhance the thermoelectric power factor in bulk materials. *Phys. Rev. B - Condens. Matter Mater. Phys.* **87**, 075204 (2013).
14. Kim, H. S., Gibbs, Z. M., Tang, Y., Wang, H. & Snyder, G. J. Characterization of Lorenz number with Seebeck coefficient measurement. *APL Mater.* **3**, 041506 (2015).
15. *Sintering and Grain Growth. In: Ceramic Materials*. *Ceramic Materials* (Springer, New York, NY, 2007). doi:10.1007/978-0-387-46271-4_24
16. Wang, K., Yang, D., Wu, C., Sanghadasa, M. & Priya, S. Recent Progress in Fundamental Understanding of Halide Perovskite Semiconductors. *Prog. Mater. Sci.* 100580 (2019). doi:10.1016/J.PMATSCI.2019.100580
17. Leguy, A. M. A. *et al.* Experimental and theoretical optical properties of methylammonium lead halide perovskites. *Nanoscale* **8**, 6317–6327 (2016).
18. Saidaminov, M. I. *et al.* High-quality bulk hybrid perovskite single crystals within minutes by inverse temperature crystallization. *Nat. Commun. 2015 61* **6**, 1–6 (2015).
19. Chen, Z. *et al.* Thin single crystal perovskite solar cells to harvest below-bandgap light absorption. *Nat. Commun. 2017 81* **8**, 1–7 (2017).
20. Dong, Q. *et al.* Lateral-Structure Single-Crystal Hybrid Perovskite Solar Cells via

Piezoelectric Poling. *Adv. Mater.* **28**, 2816–2821 (2016).
21. Miyasaka, T. Perovskite Photovoltaics: Rare Functions of Organo Lead Halide in Solar Cells and Optoelectronic Devices. *Chem. Lett.* **44**, 720–729 (2015).
22. Song, Y. *et al.* Efficient lateral-structure perovskite single crystal solar cells with high operational stability. *Nat. Commun. 2020 111* **11**, 1–8 (2020).
23. Hoque, M. N. F. *et al.* Polarization and Dielectric Study of Methylammonium Lead Iodide Thin Film to Reveal its Nonferroelectric Nature under Solar Cell Operating Conditions. *ACS Energy Lett.* **1**, 142–149 (2016).
24. Bai, Z. Q. & Liu, Z. W. A broadband photodetector based on Rhodamine B-sensitized ZnO nanowires film. *Sci. Reports 2017 71* **7**, 1–8 (2017).
25. Tang, B. *et al.* UV-SWIR broad range photodetectors made from few-layer α-In_2Se_3 nanosheets. *Nanoscale* **11**, 12817–12828 (2019).
26. Lee, B. *et al.* Air-stable molecular semiconducting iodosalts for solar cell applications: Cs_2SnI_6 as a hole conductor. *J. Am. Chem. Soc.* **136**, 15379–15385 (2014).
27. Skoug, E. J. & Morelli, D. T. Role of lone-pair electrons in producing minimum thermal conductivity in nitrogen-group chalcogenide compounds. *Phys. Rev. Lett.* **107**, 235901 (2011).
28. Mukhopadhyay, S. *et al.* Two-channel model for ultralow thermal conductivity of crystalline Tl_3VSe_4. *Science* **360**, 1455–1458 (2018).
29. Sajjad, M., Mahmood, Q., Singh, N. & Andreas Larsson, J. Ultralow Lattice Thermal Conductivity in Double Perovskite Cs_2PtI_6: A Promising Thermoelectric Material. *ACS Appl. Energy Mater.* **3**, 11293–11299 (2020).
30. Saparov, B. *et al.* Thin-Film Deposition and Characterization of a Sn-Deficient Perovskite Derivative Cs_2SnI_6. *Chem. Mater.* **28**, 2315–2322 (2016).
31. Zhu, W. *et al.* Deciphering the degradation mechanism of the lead-free all inorganic perovskite Cs_2SnI_6. *npj Mater. Degrad.* **3**, 2–8 (2019).
32. Liu, D. *et al.* Strain analysis and engineering in halide perovskite photovoltaics. *Nat. Mater. 2021 2010* **20**, 1337–1346 (2021).
33. Xue, D. J. *et al.* Regulating strain in perovskite thin films through charge-transport layers. *Nat. Commun. 2020 111* **11**, 1–8 (2020).
34. Fang, H. *et al.* A self-powered organolead halide perovskite single crystal photodetector driven by a DVD-based triboelectric nanogenerator. *J. Mater. Chem. C* **4**, 630–636 (2016).
35. Zhang, Y., Liu, Y., Li, Y., Yang, Z. & Liu, S. Perovskite $CH_3NH_3Pb(Br_xI_{1-x})_3$ single crystals with controlled composition for fine-tuned bandgap towards optimized optoelectronic applications. *J. Mater. Chem. C* **4**, 9172–9178 (2016).
36. Ding, J. *et al.* A self-powered photodetector based on a $CH_3NH_3PbI_3$ single crystal with asymmetric electrodes. *CrystEngComm* **18**, 4405–4411 (2016).
37. Zhang, Y. *et al.* Ultrasensitive Photodetectors Based on Island-Structured $CH_3NH_3PbI_3$ Thin Films. *ACS Appl. Mater. Interfaces* **7**, 21634–21638 (2015).
38. Ye, T. *et al.* Ultra-high Seebeck coefficient and low thermal conductivity of a centimeter-sized perovskite single crystal acquired by a modified fast growth method. *J. Mater. Chem. C* **5**, 1255–1260 (2017).
39. Haque, M. A., Nugraha, M. I., Paleti, S. H. K. & Baran, D. Role of Compositional Tuning on Thermoelectric Parameters of Hybrid Halide Perovskites. *J. Phys. Chem. C* **123**, 14928–14933 (2019).

40. Mettan, X. *et al.* Tuning of the Thermoelectric Figure of Merit of $CH_3NH_3MI_3$ (M=Pb,Sn) Photovoltaic Perovskites. *J. Phys. Chem. C* **119**, 11506–11510 (2015).
41. Wu, P., Xiong, Y., Sun, L., Xie, G. & Xu, L. Enhancing thermoelectric performance of the $CH_3NH_3PbI_3$ polycrystalline thin films by using the excited state on photoexcitation. *Org. Electron.* **55**, 90–96 (2018).
42. Long, X., Pan, Z., Zhang, Z., Urban, J. J. & Wang, H. Solvent-free synthesis of organometallic halides $CH_3NH_3PbI_3$ and $(CH_3NH_3)_3Bi_2I_9$ and their thermoelectric transport properties. *Appl. Phys. Lett.* **115**, 072104 (2019).
43. Li, W. *et al.* Bismuth Telluride/Half-Heusler Segmented Thermoelectric Unicouple Modules Provide 12% Conversion Efficiency. *Adv. Energy Mater.* **10**, 2001924 (2020).
44. Pan, Y. *et al.* $Mg_3(Bi,Sb)_2$ single crystals towards high thermoelectric performance. *Energy Environ. Sci.* **13**, 1717–1724 (2020).
45. Henkels, H. W. Thermoelectric Power and Mobility of Carriers in Selenium. *Phys. Rev.* **77**, 734 (1950).
46. Pisoni, A. *et al.* Ultra-Low Thermal Conductivity in Organic–Inorganic Hybrid Perovskite $CH_3NH_3PbI_3$. *J. Phys. Chem. Lett.* **5**, 2488–2492 (2014).
47. Elbaz, G. A. *et al.* Phonon Speed, Not Scattering, Differentiates Thermal Transport in Lead Halide Perovskites. *Nano Lett.* **17**, 5734–5739 (2017).
48. Ge, C. *et al.* Ultralow Thermal Conductivity and Ultrahigh Thermal Expansion of Single-Crystal Organic-Inorganic Hybrid Perovskite $CH_3NH_3PbX_3$ (X = Cl, Br, I). *J. Phys. Chem. C* **122**, 15973–15978 (2018).
